# Reduced *SH3RF3* May Protect Against Alzheimer's Disease by Lowering Microglial Pro‐Inflammatory Responses via Modulation of JNK and NFkB Signaling

**DOI:** 10.1002/glia.70165

**Published:** 2026-05-27

**Authors:** Ronak Patel, Rong Cheng, Christopher L. Cardona, Ellen Angeles, Gunjandeep Singh, Sabrina Miller, Archana Ashok, Andrew F. Teich, Angel Piriz, Aleyda Maldonado, Ivonne Z. Jiménez‐Velázquez, Richard Mayeux, Joseph H. Lee, Andrew A. Sproul

**Affiliations:** ^1^ Taub Institute for Research on Alzheimer's Disease and the Aging Brain Columbia University Irving Medical Center (CUIMC) New York New York USA; ^2^ Department of Neurology CUIMC New York New York USA; ^3^ Department of Pathology and Cell Biology CUIMC New York New York USA; ^4^ East Side Community School New York New York USA; ^5^ G.H. Sergievsky Center CUIMC New York New York USA; ^6^ Department of Internal Medicine, Geriatric Division University of Puerto Rico School of Medicine San Juan Puerto Rico USA; ^7^ Department of Epidemiology CUIMC New York New York USA

**Keywords:** Alzheimer's disease, Aβ, inflammation, JNK, microglia, NFκB, *POSH2*, *PSEN1*, *SH3RF3*

## Abstract

Understanding how high‐risk individuals are protected from Alzheimer's disease (AD) may illuminate potential therapeutic targets. We identified protective genetic variants in *SH3RF3*/*POSH2* that delayed the onset of AD among individuals carrying the *PSEN1* ^G206A^ mutation. *SH3RF3* acts as a JNK pathway scaffold and activates NFκB signaling. While the effects of *SH3RF3* knockdown in human neurons were subtle, including decreased pTau S422, knockdown in human microglia significantly reduced inflammatory cytokines in response to either a viral mimic or oAβ42. This was associated with reduced activation of JNK and NFκB pathways in response to these stimuli. Pharmacological inhibition of JNK or NFκB signaling phenocopied *SH3RF3* knockdown. We also found *PSEN1* ^G206A^ microglia had a reduced inflammatory response to oAβ42. Thus, further reduction of microglial inflammatory responses in *PSEN1* ^G206A^ mutant carriers by protective variants in *SH3RF3* might reduce the link between amyloid and neuroinflammation to subsequently delay the onset of AD.

## Graphical abstract

- SNPs in *SH3RF3* decrease the age of onset of Alzheimer's disease (AD).
- Reduced SH3RF3 blunts human microglial JNK and NFkB signaling and downstream inflammatory cytokine production, which may partially explain how *SH3RF3* SNPs protect against AD.

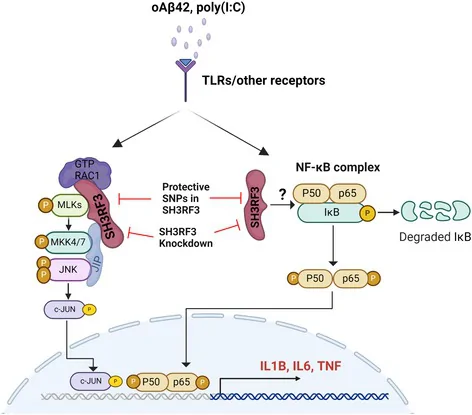

## 1 Introduction

The amyloid hypothesis (Selkoe and Hardy 2016) has largely guided therapeutic approaches to treat Alzheimer's disease (AD) the past few decades. Although use of monoclonal antibodies to target Aβ removal has recently gained traction and FDA approval, clinical benefits are modest and have risks such as ARIA‐E/H (amyloid‐related imaging abnormalities‐edema/hemorrhage) (van Dyck et al. 2023; Soderberg et al. 2023). Thus, additional therapeutic strategies are necessary. Instead of targeting neuropathology directly, an alternative tactic is to better understand why some individuals are resilient to AD and then attempt to mimic this resistance via pharmacological manipulation or other interventions. One robust approach to understand resilience is the study of genetic factors which modify the age at onset of AD in high‐risk populations such as presenilin‐1 (*PSEN1*) mutation carriers, which is sufficient to cause early‐onset AD (EOAD).

To date, much of the genetic work has been focusing on identifying risk variants, and a limited number of studies have made efforts to identify protective genetic factors (Lee et al. 2015; Lopera et al. 2023). As commented by Schwartz and colleagues (Schwartz et al. 2017), protective genetic modifiers are likely to provide important insights into our understanding of the disease mechanisms of AD in the era of precision medicine. Our previous examination of phenotypic variation in the families with the G206A mutation in the *PSEN1* gene revealed that carriers of this mutation may display age at onset ranging from the 40s to 80s even within the same family, and environmental risk factors explained little of the phenotypic variance. Our linkage analysis localized 2 loci, 2q13 and 4q35, that were likely to be associated with variation in age at onset of AD. Further examination of 2q13 identified *SH3RF3*/*POSH2* (*plenty of SH3 domains 2*) as one of the potential genetic modifiers that ameliorate the effects of the G206A mutation in *PSEN1* (Lee et al. 2015). Among *PSEN1* ^G206A^ carriers, *SH3RF3* was associated with a delay in age at onset of symptoms by 9.3 years. We further showed that variants in *SH3RF3* were associated with 0.8–2.9 years in delay in age at onset of symptoms in late onset AD (LOAD). This suggests that *SH3RF3* may serve as a genetic modifier in both early‐onset AD (EOAD) and late‐onset AD (LOAD), but the protective effect, represented by the mean years of accelerated or delayed onset is likely to be smaller in LOAD when compared with EOAD. Along with *SH3RF3*, we identified six other candidate genetic modifiers in 2q13 and 4q35 that were nominally significant in both EOAD and LOAD, suggesting that modifiers that were discovered in EOAD may be generalizable to LOAD, the common form of AD.

We have focused this study on better understanding of the mechanisms of how *SH3RF3* may protect against AD. In addition to our study showing effects on AD risk (Lee et al. 2015), *SH3RF3* has also been identified by causal inference analysis as a key microglial‐expressed regulator of late‐onset AD (LOAD) (Park and Kellis 2021) and in a genome‐wide association study (GWAS) focused on HIV‐associated neurocognitive disorder (HAND) (Jia et al. 2017). SH3RF3 functional domains include 4 protein–protein interaction SH3 domains, a novel Rac1 binding domain that only binds the active GTP‐bound version, and a RING domain which gives SH3RF3 E3 ligase activity to target itself (and potentially other proteins) for proteasomal degradation. There are 3 *SH3RF*/*POSH* family members, which also include *SH3RF1*/*POSH* which has the same domain structure as *SH3RF3*, and *SH3RF2*/*POSHER* (*POSH‐eliminating RING protein*), which is missing the 4^th^ SH3 domain. Both *SH3RF1* and *SH3RF3* have been identified as scaffold proteins promoting JNK (c‐Jun N‐terminal kinase) signaling as well as having the ability to activate NFκB signaling by an unknown mechanism when overexpressed (Karkkainen et al. 2010; Tapon et al. 1998; Zhang et al. 2020). The ability to act as a JNK scaffold has best been described for SH3RF1 (Xu et al. 2003; Kukekov et al. 2006), which directly binds GTP‐Rac1 and MAP3Ks (*mitogen‐activated protein kinase kinase kinase*) such as MLK3 (*mixed lineage kinase 3*) as well as JIP (*JNK‐interacting protein*) family member scaffolds. JIP scaffolds, in turn, directly bind MAP2Ks (mitogen‐activated kinase kinase; MKK4/7) and JNKs, which then phosphorylate c‐Jun to activate the AP‐1 transcription factor dimer (see also *Graphical Abstract*). On the other hand, SH3RF2/POSHER was reported to target SH3RF1/POSH for proteasomal degradation and interfere with its ability to promote JNK signaling (Wilhelm et al. 2012).

JNK and NFκB signaling pathways have both been shown to be strongly associated with AD pathogenesis and are activated by Aβ and upregulated in AD brains (Kim and Choi 2010; Yarza et al. 2015; Sun et al. 2022; Shi et al. 2016). In neurons, JNK signaling has been shown to directly phosphorylate APP at T688 to promote amyloidogenic processing (Lee et al. 2003; Colombo et al. 2009; Yoon et al. 2012) and tau at multiple residues (Yoshida et al. 2004; Reynolds et al. 1997), increase BACE1 transcription (Tamagno et al. 2009), and is required for cell death of murine neurons from oligomeric Aβ42 (Troy et al. 2001). In microglia, JNK signaling has been reported to mediate Aβ‐driven cytokine production (Dong et al. 2016), and has its activity increased by reduced *TREM2* expression (Zhong et al. 2017; Ruganzu et al. 2022). Interestingly, NFκB signaling has also been reported to drive *BACE1* transcription and thus amyloidogenic processing (Chen et al. 2012). The major pathogenic role of NFκB is that of a key driver of inflammatory responses such as the production of pro‐inflammatory cytokines and reactive oxygen species (ROS) in glial cells, which is both detrimental in itself to neurons and promotes amyloid and tau pathology as well as damage to the blood brain barrier (Sun et al. 2022; Shi et al. 2016). Importantly, a recent study using human induced pluripotent stem cell (hiPSC)‐derived microglia (iMGLs) xenotransplanted into an AD mouse model (APP^NL‐G‐F^) demonstrated that human microglia have unique cytokine response modules (CRMs) in response to amyloid not seen in murine microglia (Mancuso et al. 2024). CRMs are distinct from the DAM response by pseudotime analysis, are associated with NFκB signaling and pro‐inflammatory cytokine production, and include *SH3RF3* as a member (CRM2). Therefore, we postulated that reduced *SH3RF3* expression or function would be protective against AD by mitigating extended activation of both JNK and NFκB pathways and tested this hypothesis in both hiPSC‐derived neuronal and microglial (iMGLs) models.

## 2 Methods

### 2.1 Genetic Association Analysis

Extending our earlier work (Lee et al. 2015), we performed WGS on a larger set of Caribbean Hispanic families with at least one *PSEN1* ^G206A^ carrier. The study protocol was approved by the Institutional Review Board of Columbia University and the University of Puerto Rico, School of Medicine, and written informed consent was provided by all study participants. To confirm the findings from our discovery dataset and generalize to more common forms of AD (i.e., familial and sporadic LOAD), we examined 1946 singletons and 2114 family members of the familial late onset AD (LOAD), comprising 4060 Caribbean Hispanic elderly, primarily from the Dominican Republic (EFIGA (Lee et al. 2004)). In addition, we examined 2058 Hispanic elderly with primarily sporadic AD (WHICAP (Tang et al. 1998)).

#### 2.1.1 Whole Genome Sequencing (WGS)

To enhance the resolution of the *SH3RF3* gene, we first performed WGS on about two‐thirds of the family members (*n* = 337) from a total of 505 family members in 72 families. The genome sequencing used 1 μg of DNA, an Illumina PCR‐free library protocol, and was sequenced on the Illumina HiSeq platform at the NY Genome Center. We assessed sex mismatches and unexpected familial relationships prior to analyses. Genomes were sequenced to a mean coverage of 30×. Sequence data analysis was performed using an automated analysis pipeline that matched the Centers for Common Disease Genomics (CCDG) and TOPMed recommended best practices. Briefly, sequencing reads were aligned to the human reference, hs38DH, using Burrows‐Wheeler Alignment—Maximal Exact Matches (BWA‐MEM) version 0.7.15. Variant calling was performed using the Genome Analysis Toolkit (GATK) best practices. Variant filtration was performed using Variant Quality Score Recalibration (VQSR) at tranche 99.6%, which identifies annotation profiles of variants that could be called with high confidence and assigns a score Variant Quality Score Log‐Odds (VQSLOD) to each variant. Variants passing the VQSR threshold were further filtered out for sample missingness (> 2%), depth of coverage (DP) < 10, and genotype quality (GQ) > 20. We then annotated high‐quality variants using Annotate Variation (ANNOVAR). Specifically, variants were annotated for population‐level frequency using Genome Aggregation Database (gnomAD), in silico function using Variant Effect Predictor (VEP), and variant conservation using Combined Annotation Dependent Depletion (CADD) score.

#### 2.1.2 Genome‐Wide Association (GWA) Analysis

For approximately one‐third of the family members, we performed GWA using the Illumina Human Hap 650k and Illumina 1M arrays and Illumina Infinium General Screening Array V2 or V3 (Illumina, San Diego, CA, USA) with added disease markers at the Center for Applied Genomics at Children's Hospital of Pennsylvania. Genotype calling was done using Illumina's GenomeStudio v2.0.5 with the Genotyping module; in total, 664,337 QCed variants were genotyped for 430 study participants from *PSEN1* ^G206A^ Cohort, and 572,421 QCed variants for 4060 samples of EFIGA cohort and 532,147 QCed variants for 2058 samples of WHICAP cohort.

#### 2.1.3 Imputation

To increase the coverage of the genome for those with GWAS data only, we performed imputation on all autosomal chromosomes using the TOPMed Imputation Server (https://imputation.biodatacatalyst.nhlbi.nih.gov/), and the TOPMed Reference Panel (Version R2) built on whole genome sequencing data from 97,256 individuals and 308,107,085 genetic variants of diverse ancestral and ethnic backgrounds (Das et al. 2016). This TOPMed panel included 487 Puerto Ricans, 1062 Costa Ricans, and 504 Mexicans to enhance the relevance of the reference panel. A previous study had shown that imputation in these Puerto Ricans was accurate (Sariya et al. 2019). We included all variants with imputation quality of at least R (van Dyck et al. 2023) > 0.30 for further analysis.

Prior to analysis, we conducted the standard quality control checks, including checking of the reported family relationships using the software PREST (https://www.utstat.utoronto.ca/sun/Software/Prest/), Hardy–Weinberg equilibrium, and genotyping rate. The details of the quality control procedures are presented elsewhere (Sun et al. 2017).

#### 2.1.4 Statistical Analysis

We first examined the relationship between genetic variants and age at last follow up (i.e., age at onset for affected family members and age at last examination for unaffected family members) using 337 family members with WGS data. We performed age at onset analysis using the generalized linear mixed model as implemented in GMMAT (version 1.4.2) (Chen et al. 2016). The GMMAT model adjusted for sex, *APOE4* genotype, *PSEN1* ^G206A^ status, affection status, principal components (PC1–PC3), and genetic relationship matrix (GRM). PC1 to PC3 were included to adjust for genetic background, and GRM was included to take into account relatedness among family members.

Subsequently, we re‐analyzed the data after adding 168 family members who lacked WGS data but had imputed genotype data. Our earlier work showed that imputation in Caribbean Hispanic families was accurate (Sariya et al. 2019). We applied this 2‐stage approach to gain finer resolution, while maintaining cost‐effectiveness. Even though the WHICAP study was not a family‐based study, we kept the genetic relationship matrix (GRM) as a covariate to adjust for a small number of pairwise relationships that differed from IBD of 0.

### 2.2 hPSC Maintenance and Differentiation Into Neurons and Microglia‐Like Cells (iMGLs)

The FA0000010 (FA10) hiPSC line was acquired from RUCDR, and the IMR90 (cl.4) hiPSC line (Yu et al. 2007) and the H9 (WA09) hESC line from WiCell. All cultures were grown in the presence of penicillin/streptomycin unless noted otherwise (ThermoFisher). Undifferentiated hPSCs were maintained in StemFlex media (ThermoFisher) on reduced growth factor Cultrex BME (Biotechne), and routinely split 1–2 times a week with ReLeSR (STEMCELL Technologies) without ROCKi (Y‐27632, Selleck Chemicals) as described previously (Song et al. 2021). Generation and transdifferentiation of permanent transgene hPSCs (Ngn2/rtTA) into excitatory neurons were described in detail previously (Song et al. 2021). In brief, undifferentiated H9 hPSCs with permanent Ngn2/rtTA transgenes (via puromycin selection) were plated in the presence of 10 μM Y‐27632 (ROCKi) on PEI/Laminin‐coated surfaces at 55,000–60,000 cells/cm^2^ (200 k/well of a 12 well) in N2/B27 medium supplemented with 1 μg/mL doxycycline (Sigma‐Aldrich), 10 ng/mL BDNF (R&D systems), 10 ng/mL NT3 (R&D systems), and 2 μM AraC (cytarabine, Tocris) which was added for the first 7 days of differentiation as needed to deplete non‐neuronal cells from culture. At day 7, cultures were switched to Brainphys (N2/SM1) neuronal differentiation medium (STEMCELL Technologies) supplemented with 1 μg/mL doxycycline, 10 ng/mL BDNF, 10 ng/mL NT3 with half medium change every other day for the duration of differentiation. Brainphys neuronal differentiation medium was further supplemented with 10% mouse astrocyte conditioned media (ACM; ScienCell) from d14 until samples are collected on d28.

hiPSCs were differentiated into iMGLs as described previously with minor adaptations (Abud et al. 2017; McQuade et al. 2018). hiPSCs were differentiated into hematopoietic precursor cells (HPCs) using the STEMdiff Hematopoietic Kit (STEMCELL Technologies) largely as per manufacturer's instructions. In brief, on day −1 iPSCs were detached with ReLeSR and passaged to achieve a density of 1–2 aggregates/cm^2^ of 100–150 cells. Multiple densities were plated in parallel. On day 0, colonies of appropriate density were switched to Medium A from the STEMdiff Hematopoietic Kit to initiate HPC differentiation. On day 3, cells were switched to Medium B with a full media change and fed again with a full media change on day 5. Cells remained in Medium B for the rest of the HPC differentiation period with Medium B overlay feeds every other day. HPCs were collected 3 independent times by gently removing the floating population with a serological pipette at days 11, 13, and 15 (or days 12, 14, and 16). HPCs were either cryopreserved in 45% Medium B, 45% knockout serum replacement (ThermoFisher), and 10% DMSO and stored in liquid nitrogen or directly plated for iMGL induction. Pilot differentiations showed a majority of HPCs were CD43^+^ as measured by flow cytometry (> 90%). HPCs were terminally differentiated by plating 35,000–60,000 cells/cm^2^ HPCs on reduced growth factor Cultrex BME in microglia medium (DMEM/F12, 2× insulin‐transferrin‐selenite, 2× B27, 0.5× N2, 1× glutamax, 1× non‐essential amino acids, 400 mM monothioglycerol, and 5 mg/mL human insulin (ThermoFisher)) freshly supplemented with 100 ng/mL IL‐34, 25 ng/mL M‐CSF (R&D Systems), and 50 ng/mL TGFβ1 (STEMCELL Technologies) for every other day until day 24. For most differentiations, differentiating cells were “split” by taking remaining non‐adherent cells and plating onto a fresh Cultrex‐coated well of the same format (1:1). On day 25, 100 ng/mL CD200 (Bon Opus Biosciences) and 100 ng/mL CX3CL1 (R&D Systems) were added to microglia medium to mimic a brain‐like environment. For all experiments in this study, iMGLs were treated between days 28 and 35 (post‐HPC) and samples were collected on days 34–36. The majority of iMGL experiments were generated from FA10 hiPSCs unless stated otherwise.

### 2.3 *SH3RF3* Knockdown in Neurons and iMGLs

*SH3RF3* knockdown was performed using Accell SMARTpool siRNAs targeting *SH3RF3*, with Accell human non‐targeting pool siRNA used as a negative control (Dharmacon/Horizon Discovery). siRNAs were resuspended and delivered as per manufacturer's instructions. In brief, 50 nmol siRNA was resuspended using 1× siRNA buffer at 100 μM stock concentration and directly delivered at 1 μM concentration in cell culture media (at day 21 for neurons, day 28 for iMGLs). Knockdown was performed for 7 days with media overlay at days 3 and 5. RNA, protein lysates, and conditioned media (CM) were collected from neurons at day 28 and iMGLs at day 35.

### 2.4 Treatment of Neurons and iMGLs With Chemical Inhibitors and Inflammatory Stimuli

On day 28, neurons were treated with okadaic acid or vehicle for 3 h before lysing for protein and conditioned media. In some experiments, iMGLs were pre‐treated with chemical inhibitors or DMSO vehicle for 2 h before inflammatory stimuli, which included CEP‐1347 (100 nM; Tocris), IKK16 (1 μM; Tocris), and NSC23766 (50 μM; EMD Millipore). iMGLs were then treated with either the dsRNA viral mimic poly(I:C) at (25 μg/mL) (Lafaille et al. 2012), oligomerized Aβ42 (5 μM; rPeptide), or vehicle control (dH_2_O or F12 medium respectively). Recombinant Aβ42 was oligomerized as described previously (Kim et al. 2023), and validated via Coomassie blue staining and western blot using the 6E10 antibody (1:1000; BioLegend).

### 2.5 qPCR

RNA was extracted using the RNeasy Plus Mini Kit (Qiagen) and resuspended in 30 μL of RNAse‐free water. RNA quantity and quality were assessed with a Nanodrop One (ThermoFisher). For qPCR, 100 ng of RNA was transcribed into complementary DNA Maxima First Strand cDNA Synthesis Kit (ThermoFisher). Gene expression was assessed using FAST SYBR Green Master Mix (ThermoFisher) using primer sets for target genes on a QuantStudio 7 Flex system (Applied Biosystems) and Bio‐Rad CFX Opus 384. Gene expression for target genes was normalized to *GAPDH* and fold changes were calculated as ΔΔC_t_.

### 2.6 Amyloid‐β42 Monomerization, Oligomerization, and Characterization

Amyloid‐β42 was monomerized, stored, and later oligomerized as previously described (Kim et al. 2023; Stine Jr. et al. 2003). In brief, Aβ42 (rPeptide, 1 mg) vial was allowed to equilibrate to room temperature. Peptides were resuspended in ice‐cold Hexafluoroisopropanol (Sigma‐Aldrich) to achieve a 1 mM concentration directly in a vacuum‐sealed vial using a Hamilton syringe. The suspension was divided into 8 equal aliquots by volume using a Hamilton syringe into low protein binding Eppendorf tubes, incubated at room temperature for 2 h, and dried on speedvac at 800 *g*. Upon drying, monomerized (Aβ42) films can be stored at −80°C for months to years in sealed tubes. For generating oligomers, Eppendorf tubes containing Aβ42 peptide films were equilibrated to room temperature, resuspended in DMSO to achieve a 5 mM concentration, and sonicated at 17°C for 10 min for complete resuspension. Oligomerization was induced by adding phenol‐red free F12 to achieve a final concentration of 100 μM and incubating at 4°C for 24 h and was used for experiments the following day. 200 ng of oligomers were prepared in the absence of reducing reagents and run on a 4%–20% bis‐tris gel in MES running buffer. Gels were transferred onto a 0.2 μm nitrocellulose membrane to probe against amyloid‐β (6E10 antibody, BioLegend).

### 2.7 Western Blotting

iMGLs or neurons were harvested using RIPA Lysis Buffer (ThermoFisher) supplemented with Halt protease and phosphatase inhibitors single‐use cocktail (ThermoFisher) on ice. 10 μg of lysates and 1× Laemmli sample buffer/5% BME were boiled at 95°C for 10 min. SDS‐PAGE was performed by loading samples on Bolt 4%–12% gradient Bis‐Tris gels (ThermoFisher) using Bolt MOPS buffer (ThermoFisher). For western blotting, nitrocellulose membranes were first blocked with Superblock TBS buffer/0.1% Tween 20 (ThermoFisher) for 1 h, and then incubated overnight at 4°C with the following primary antibodies diluted in Superblock/0.1% Tween 20: p‐JNK (1:1000; Cell Signaling Technology), total‐JNK(1:1000; Cell Signaling Technology), phospho‐p‐50/p105 (1:1000; Cell Signaling Technology), total p50/p105 (1:1000; Cell Signaling Technology), phospho‐Tau Ser422 (1:1000; Sigma), phospho‐Tau Thr217 (1:1000; Abcam), and GAPDH (1:5000; ThermoFisher). After 3 washes with TBS/0.1% Tween 20, membranes were then incubated with the appropriate HRP‐conjugated secondary antibody (1:5000; ThermoFisher) for 1 h and washed 3 more times with TBS/0.1% Tween 20. Pierce ECL western blotting substrate (ThermoFisher) or super signal west pico plus chemiluminescent substrate (ThermoFisher) was used to develop chemiluminescence and blots were imaged using the Bio‐Rad Chemidoc MP system.

### 2.8 Multiplex ELISA for Aβ or lL‐1β, IL6, and TNFα

Conditioned media (CM) from neurons or iMGLs were first spun at 1000 rpm for 3 min, and SNF was aliquoted and stored at −80°C. CM was diluted 1:2 in diluent 35 and processed for ELISA as per the manufacturer's instructions. The Aβ peptide panel 1 ELISA kit and custom cytokine kits U‐Plex biomarker group 1 (human) ELISA kit were from Mesoscale Discovery (MSD Quickplex SQ120). Analyte concentrations (pg/mL) were calculated as per manufacturer's instruction.

### 2.9 Immunocytochemistry

iMGLs were plated on low growth factor Cultrex‐coated 24‐well plates, cultured for 35 days and fixed with 4% paraformaldehyde for 20 min on ice. After fixation, cells were blocked with Superblock blocking buffer in PBS/0.1% Triton‐X for 1 h and then incubated with the following primary antibodies overnight at 4°C: α‐CD11b (1:100; BD Biosciences) and α‐Iba1 (1:200; Wako). Next day, cells were washed thrice with PBS/0.1% Triton‐X and then incubated with corresponding Alexa Fluor‐conjugated secondary antibodies (1:500; ThermoFisher) at room temperature for 1 h. Cells were washed thrice with PBS/0.1% Triton‐X and imaged using an ECHO revolve 2 microscope. DAPI (ThermoFisher) was used as a nuclear stain.

### 2.10 *PSEN1* ^G206A^ Knock‐In via CRISPR/Cas9

The G206A mutation was knocked into both alleles of *PSEN1* in IMR90 cl.4 iPSCs using CRISPR/Cas9 as has been done previously for the APP^Lon^ mutation (Sun et al. 2019). In brief, sgRNAs targeting exon 7 of *PSEN1* were designed (Deskgen.com) and subcloned into MLM3636 vector, a gift from Keith Joung (Addgene plasmid # 43860; http://n2t.net/addgene:43860; RRID: Addgene_43860). sgRNAs were tested for efficacy in HEK293 (ATCC) using a genomic cleavage kit (GeneArt, ThermoFisher). The best sgRNA was used for editing and had the sequence: TTCACTGGAAAGGTCCACTT. The ssDNA HDR template was designed with a silent CRISPR blocking mutation at the PAM site which added a de novo EcoR V restriction site in addition to the G206A mutation. Introduced changes are shown below in bolded under case in the HDR sequence. All oligos for genome editing were ordered from Integrated DNA technologies. HDR: mutant sequence: GAAGTGTTTAAAACCTATAACGTTGCTGTGGACTACATTACTGTTGCACTCCTGATCTGGAATTT TG**c**TGTGGTGGGAATGAT**a**TC**g**ATTCACTGGAAAGGTCCACTTCGACTCCAGCAGGCATATC, eSpCas9 (1.1), a gift from Feng Zhang (Slaymaker et al. 2016) (Addgene plasmid # 71814; http://n2t.net/addgene:71814; RRID: Addgene_71814), ssDNA HDR template, sgRNA and GFP plasmid (Lonza) were electroporated (Lonza nucleofector) into feeder‐free iPSCs, followed by cell sorting on GFP signal and plating at low density on MEFs (MTI‐GlobalStem). Individual clones were manually picked and transferred to a 96‐well format plate, which was split into duplicate plates and high and low density. gDNA was collected from high density plates as previously described (Paquet et al. 2016). For each clone, exon 7 of *PSEN1* was amplified and screened by restriction digest by EcoR V (New England Biolabs). Sanger sequencing was used to confirm the mutation (Azenta), and successful clones were expanded and banked. *PSEN1* ^G206A^ knock‐in lines were analyzed and demonstrated to have a normal karyotype (Cell Line Genetics) and to be bona fide homozygous knock‐ins rather than hemizygous by a quantitative genotyping PCR method (Weisheit et al. 2020). Cl.95 was used in the present study.

### 2.11 Statistics

All experiments were repeated independently and information about number of repeated experiments, replicates, error bars, sample size, the statistical test used, and measure of significance are provided in corresponding figure legends. Independent wells were considered biological rather than pure technical replicates due to 4‐week long differentiations in which small stochastic differences at plating could significantly impact cells when assayed. Independent experiments refer to independent differentiations. Statistical analyses were performed in Prism software version 9.0 (GraphPad). Unpaired *t*‐test was used for comparing the means of two groups. For experiments with more than 2 groups, data were analyzed using a mixed‐effects model with treatment group and differentiation as fixed factors and replicates as a random effect. The main effect of the treatment group, averaged across differentiations, was assessed, followed by Tukey's multiple comparisons test, as has been done previously by others (Terzioglu et al. 2025; Lish et al. 2025).

*Chart of All Reagents*:

**Table:** 

Reagent or resource	Source	Identifier
*Antibodies*		
p‐SAPK/JNK (T138/Y185) (81E11)	Cell Signaling Technology	cat.# 4668S; RRID: AB_823588
SAPK‐JNK	Cell Signaling Technology	cat.# 9252S; RRID: AB_2250373
p‐NF‐kappaβ P105 (S932) (18E6)	Cell Signaling Technology	cat.# 4806S; RRID: AB_2282911
NF‐kappaβ P105/P50 (SD10D11)	Cell Signaling Technology	cat.# 13681S; RRID: AB_2798292
p‐c‐JUN (S73) (D47G9)	Cell Signaling Technology	cat.# 3270S; RRID: AB_2895041
c‐JUN (L70B11)	Cell Signaling Technology	cat.# 2315S; RRID:AB_490780
CD45 Alexa fluor 647 conjugated	BioLegend	cat.# 304056; RRID: AB_2564155
CD11b Alexa fluor 488 conjugated	BioLegend	cat.# 301318; RRID: AB_493017
IBA1	FUJIFILM	cat.# 019‐19741; RRID: AB_839504
P2RY12	Millipore‐Sigma	cat.# HPA014518; RRID: AB_2669027
TREM2	Cell Signaling Technology	cat.# 91068S; RRID: AB_2721119
Anti‐Aβ 6E10	BioLegend	cat.# SIG‐39320; RRID: AB_2715854
Phospho‐APP (Thr668)	Cell Signaling Technology	cat.#6986; RRID:AB_10831197
APP (Y188)	Abcam	cat.# ab_32136; RRID_2289606
Phospho‐Tau (Thr217)	Abcam	cat.#ab_192665; RRID_2139718
Phospho‐Tau (ser422)	ThermoFisher	Cat.#44‐764G; RRID_AB_2533748
Total Tau	Dako	Cat.# A0024; RRID_AB_10013724
GAPDH	ThermoFisher	cat.# MA1‐16757; RRID: AB_568547
*Chemicals, peptides, and recombinant proteins*		
IL34	R&D	cat.# 5265‐IL‐010/CF
TGFβ	R&D	cat.# 240‐B‐010/CF
m‐CSF	R&D	cat.# 216‐MC‐010/CF
CD200	Bon‐Opus Bio	cat.# BP004
CX3CL1	R&D	cat.# 365‐FR/CF
CEP1347	Tocris	cat.# 4924
IKK16	Tocris	cat.# 2539
NSC23766 (RAC1 inhibitor)	EMD Millipore	cat.# 553502
Amyloid‐β‐1‐42	rPeptide	cat.# A‐1163‐2
Poly(I:C)	Tocris	cat.# 4287
STEMdiff Hematopoietic Kit	STEMCELL Technologies	cat.# 05310
DMEM‐F12 medium	ThermoFisher	cat.# 11320033
Non‐essential amino acids	ThermoFisher	cat.# 11140050
Insulin‐transferrin‐selenium	ThermoFisher	cat.# 41400045
Insulin	Sigma‐Aldrich	cat.# 12585014
Glutamax	ThermoFisher	cat.# 35050061
B27‐ supplements	ThermoFisher	cat.# 17504044
N2‐ supplements	ThermoFisher	cat.# 17502048
1‐Thioglycerol	Sigma‐Aldrich	cat.# M‐6145
Pen Strep	ThermoFisher	cat.# 15140122
SuperSignal West Pico PLUS	ThermoFisher	cat.# 34577
*Critical commercial assays and kits*		
Aβ peptide panel 1 ELISA kit	Mesoscale Discovery	cat. # K15200E
U‐Plex biomarker group 1 (human) ELISA kit	Mesoscale Discovery	cat. # K15067L
Genomic cleavage assay kit	GeneArt, ThermoFisher	cat. #A24372
RNeasy Plus Micro Kit	Qiagen	cat.# 74034
BCA Protein Assay Kit	Millipore Sigma	cat.# 71285‐3
*Experimental models: Cell lines*		
H9 hESC	WiCell	WA09 NIH registry
IMR90 cl.4 iPSC	WiCell	
FA0000010 (FA10) hiPSC	RUCDR	
HEK293	ATCC	
*Plasmids*		
pMLM3636	Addgene	cat.# 43860, RRID:Addgene_43860
eSpCas9 (1.1)	Addgene	cat.# 71814, RRID:Addgene_71814

*Primers*:

**Table:** 

*qPCR*	
Human qPCR primers *IL1β*	F‐ATGCACCTGTACGATCACTG
	R‐ACAAAGGACATGGAGAACACC
Human qPCR primers *IL6*	F‐CAACCTGAACCTTCCAAAGAT
	R‐ACCTCAAACTCCAAAAGACCAG
Human qPCR primers *TNFα*	F‐ACTTTGGAGTGATCGGCC
	R‐GCTTGAGGGTTTGCTACAAC
Human qPCR primers *SH3RF3*	F‐AGGACAAAGACCAAGACAAGG
	R‐GAGTCATTGAGCTCCACGTAC
Human qPCR primers *GAPDH*	F‐TGAAGGTCGGAGTCAACGGATTTGG
	R‐CATGTAGGCCATGAGGTCCACCAC
*Dharmacon Accell Pooled siRNA sequences*	
Non‐targeting	UGGUUUACAUGUCGACUAA
	UGGUUUACAUGUUUUCUGA
	UGGUUUACAUGUUUUCCUA
	UGGUUUACAUGUUGUGUGA
*SH3RF3*	CCGGAAUAAUGUAGUCGGA
	GAUGCAAACUAGACGAGAA
	CUUUAUGAUUUCGAGAUGA
	CCACUGGCCAAAGGGAUAA
*PSEN1* ^*G206A*^ *KI*	
HDR sequence	GAAGTGTTTAAAACCTATAACGTTGCTGTGGACTACATTACTGTTGCACTCCTGATCTGGAATTTTGcTGTGGTGGGAATGATaTCgATTCACTGGAAAGGTCCACTTCGACTCCAGCAGGCATATC
*PSEN1* sgRNA sequence	TTCACTGGAAAGGTCCACTT

## 3 Results

### 3.1 Identification of Additional AD Protective SNPs in *SH3RF3*

To further validate our initial discovery SNPs in *SH3RF3* (Lee et al. 2015), we significantly expanded our findings from the discovery *PSEN1* ^G206A^ families to additional cohorts to validate and to generalize to the more common forms of AD.

#### 3.1.1 Study Participants: Discovery Dataset

The present study examined 505 study participants from 78 families from Puerto Rico with at least one carrier with the G206A mutation in the *PSEN1* gene (Table 1). The study design and description of the families were provided in detail in our earlier work (Lee et al. 2015). Briefly, the mean age at onset of AD was 60.8 (SD = 10.6), but the age at onset ranged from 40 to 93, depending on the carrier status. In these families, 50.9% of the family members carried the *PSEN1* ^G206A^ mutation, and 40.6% of the family members were affected with AD where the mean age at onset was 58.1 (SD = 8.1) for *PSEN1* ^G206A^ carriers and 70.5 (SD = 13.0) for *PSEN1* ^G206A^ non‐carriers. The *APOE4* allele frequency was 17.7%. The mean years of education for these family members were 11.8 years (SD = 4.9). We performed whole genome sequencing on approximately two‐thirds (*n* = 337) of the family members, and the remaining one‐third (*n* = 168) had the genome data generated from microarray SNP data with TOPmed imputation. As shown in Table 1, mutation carriers were more likely to be subjected to whole genome sequencing than non‐carriers.

**TABLE 1:** Demographic and clinical characteristics of the PSEN1 family study participants.

Characteristics	Overall		WGSed		GWAS imputed	
	*n*	Mean or %	*n*	Mean or %	*n*	Mean or %
Number of subjects	505		337		168	
Number of families	78		72		56	
Affection status						
Affecteds	205	40.6%	159	47.2%	46	27.4%
Unaffecteds	300	59.4%	178	52.8%	122	72.6%
Number of males:females	223:282	44.2%:55.8%	149:188	44.2%:55.8%	74:94	44.0%:60.0%
Age (years)						
Age at onset for affecteds		60.8 (10.6)^a^		60.4 (10.6)		61.9 (10.5)
Age at last examination for unaffecteds		59.2 (11.7)		64.2 (9.3)		51.9 (10.9)
PSEN1‐G206A carrier status						
Carriers	257	50.9%	205	60.8%	52	31.0%
Non‐carriers	248	49.1%	132	39.2%	116	69.0%
APOE allele frequency						
E4	179	17.7%	106	15.7%	73	21.7%
E3	773	76.5%	530	78.6%	243	72.3%
E2	58	5.7%	38	5.6%	20	6.0%
Mean years of education	504	11.8 (4.9)	336	11.6 (5.0)	168	12.1 (4.9)
Affecteds	204	10.3 (5.3)	158	10.7 (5.1)	46	9.0 (5.8)
Unaffecteds	300	12.8 (4.4)	178	12.4 (4.7)	122	13.3 (3.9)

Abbreviation: SD, standard deviation.

^a^ Mean (SD).

#### 3.1.2 Allelic Association

Broadly, our multivariable allelic association model revealed that a tightly linked chromosomal region starting from 109,128,058 to 109,486,830 bp, encompassing 350 kb was significantly associated with delay in age at onset of AD as shown in Table 2. Furthermore, when stratified by the *PSEN1* ^G206A^ status, three contiguous sets of SNPs were associated with delay in age at onset of AD, with medians ranging from 5.4 to 18.6 years. In contrast, no significant delay in age at onset was observed in non‐carriers of the *PSEN1* ^G206A^ mutation. This striking difference in the observed delay in age at onset supports a possible interaction between *PSEN1* and *SH3RF3*. Specifically, a long stretch region was associated with approximately 5‐year delay in age at onset of AD from chromosome location 109,288,547–109,387,052 bp in carriers (region 2), and the low allele frequency suggested that a few haplotypes may be segregating in a limited number of families. Two other regions indicated in green—109,128,058–109,263,859 (region 1) and 109,399,707–109,486,830 (region 3) – showed significant association with stronger effect size; however, the variability was greater than that in region 2. We, however, note that regions 1 and 3 contain a synonymous mutation at 109,130,101 bp (rs77039993) and a nonsynonymous mutation at 109,449,440 bp (rs192679474) with significantly larger effect size (18.6 and 25.4, respectively). Three individuals had a synonymous variant at 109,130,101 bp, and one had a nonsynonymous variant at 109,449,440 bp.

**TABLE 2:** Allelic association with age at onset or AD or age at last follow up for *SH3RF3*: The whole genome sequencing data combined with GWAS with imputation.

2q13	BP^†^	Minor	Major	Whole genome sequencing							Combined (WGS & imputed GWAS)						Annotation function	Transcription factor
				All (*n* = 337)		Carriers (*n* = 205)			Noncarriers (*n* = 132)		All (*n* = 505)		Carriers (*n* = 257)		Noncarriers (*n* = 248)			Promoter/enhancer
				Beta	*p*	MAF	Beta	*p*	Beta	*p*	Beta	PVAL	Beta	PVAL	Beta	PVAL		Binding sites
Region 1	**109,128,058**	T	G	*11.787*	*0.01609*	0.005	*18.579*	*0.00047*	5.711	0.56084							ncRNA_exonic	**GH02J109127** (102TFs)
	**109,130,101**	**A**	**G**	***11.787***	***0.01609***	**0.005**	***18.579***	***0.00047***	**5.711**	**0.56084**							**Exonic**	**GH02J109127 (102TFs)**
	109,133,390	A	G	*11.787*	*0.01609*	0.005	*18.579*	*0.00047*	5.711	0.56084							Intronic	
	109,136,267	G	A	*11.787*	*0.01609*	0.005	*18.579*	*0.00047*	5.711	0.56084							Intronic	GH02J109133 (33TFs)
	109,138,026	CT	C	*11.787*	*0.01609*	0.005	*18.579*	*0.00047*	5.711	0.56084							Intronic	GH02J109137 (TFs)
	109,139,957	T	C	*11.787*	*0.01609*	0.005	*18.579*	*0.00047*	5.711	0.56084							Intronic	GH02J109139 (–)
	109,154,249	A	G	*9.912*	*0.00994*	0.010	*15.844*	*0.00024*	5.085	0.48623	5.261	0.188714	*12.057*	*0.00415*	−3.454	0.61468	Intronic	GH02J109154 (–)
	109,162,171	T	C	7.994	0.12258	0.002	*33.845*	*0.00067*	3.058	0.64559							Intronic	
	109,181,751	C	A	*8.888*	*0.02083*	0.010	*14.482*	*0.00095*	5.085	0.48623	3.659	0.338907	*12.082*	*0.00468*	−5.140	0.40106	Intronic	
	109,193,442	T	C	*8.888*	*0.02083*	0.010	*14.482*	*0.00095*	5.085	0.48623	3.659	0.338907	*12.082*	*0.00468*	−5.140	0.40106	Intronic	
	109,229,826	*	CTTTT	*12.098*	*0.00518*	0.005	*11.208*	*0.06635*	13.152	0.07116							Intronic	
	109,249,523	CTT	C	−1.714	0.66479	0.002	*33.777*	*0.00069*	−7.497	0.11631							Intronic	GH02J109247 (5TFs)
	**109,263,859**	T	C	10.535	0.08157	0.002	*22.889*	*0.00236*	5.711	0.56084							Intronic	
Region 2	**109,288,547**	C	T	1.481	0.30621	0.064	*4.657*	*0.00366*	−3.646	0.18246	−1.033	0.471244	2.213	0.13892	−3.715	0.11900	Intronic	GH02J109287 (5TFs)
	109,289,341	GCA	G	3.275	0.06120	0.044	*5.426*	*0.00424*	0.406	0.90785	−0.962	0.582019	2.509	0.171055	−3.970	0.19466	Intronic	GH02J109287 (5TFs)
	109,290,309	T	C	3.275	0.06120	0.044	*5.426*	*0.00424*	0.406	0.90785	−0.962	0.582019	2.509	0.171055	−3.970	0.19466	Intronic	GH02J109287 (5TFs)
	109,294,560	C	CA	3.067	0.07457	0.047	*5.012*	*0.00672*	0.406	0.90785							Intronic	
	109,294,587	A	G	3.278	0.06098	0.046	*5.301*	*0.00523*	0.703	0.84117	−0.900	0.606403	2.475	0.176696	−3.823	0.21173	Intronic	
	109,294,962	T	C	3.275	0.06120	0.044	*5.426*	*0.00424*	0.406	0.90785	−0.962	0.582019	2.509	0.171055	−3.970	0.19466	Intronic	GH02J109294 (4TFs)
	109,295,052	T	A	3.275	0.06120	0.044	*5.426*	*0.00424*	0.406	0.90785	−0.962	0.582019	2.509	0.171055	−3.970	0.19466	Intronic	GH02J109294 (4TFs)
	109,295,934	A	G	3.275	0.06120	0.044	*5.426*	*0.00424*	0.406	0.90785	−0.962	0.582019	2.509	0.171055	−3.970	0.19466	Intronic	GH02J109294 (4TFs)
	109,295,989	C	T	3.275	0.06120	0.044	*5.426*	*0.00424*	0.406	0.90785	−0.962	0.582019	2.509	0.171055	−3.970	0.19466	Intronic	GH02J109294 (4TFs)
	109,296,452	G	A	3.275	0.06120	0.044	*5.426*	*0.00424*	0.406	0.90785	−0.962	0.582019	2.509	0.171055	−3.970	0.19466	Intronic	GH02J109294 (4TFs)
	10,9312,004	A	T	1.374	0.08960	0.289	0.585	0.51054	0.774	0.60706	1.099	0.178767	0.646	0.458371	−0.185	0.88818	Intronic	
	109,334,697	G	A	*3.268*	*0.04882*	0.047	*4.344*	*0.01628*	1.644	0.61860	1.519	0.400166	*3.835*	*0.0341*	−1.333	0.68930	Intronic	GH02J109334 (9TFs)
	109,339,211	G	GA	*5.294*	*0.03320*	0.020	*7.812*	*0.00828*	2.759	0.51964							Intronic	GH02J109339 (1TF)
Region 3	109,358,421	A	G	4.252	0.34700	0.002	*33.845*	*0.00067*	−2.773	0.62693							Intronic	
	109,361,622	T	C	*7.209*	*0.03525*	0.012	*11.219*	*0.00295*	3.058	0.64559							Intronic	
	109,364,159	GT	G	*5.143*	*0.04320*	0.020	*8.149*	*0.00518*	0.654	0.89080							Intronic	
	109,381,246	T	G	4.252	0.34700	0.002	*33.845*	*0.00067*	−2.773	0.62693							Intronic	
	**109,387,052**	T	C	4.252	0.34700	0.002	*33.845*	*0.00067*	−2.773	0.62693							Intronic	GH02J109383 (17TFs)
	109,387,296	T	G	−0.906	0.23149	0.414	0.209	0.80465	−*2.981*	*0.03550*	−*1.899*	*0.01109*	−0.348	0.677144	−*3.486*	*0.00405*	Intronic	
	109,391,989	T	A	−*2.371*	*0.00913*	0.191	−0.960	0.36144	−*4.188*	*0.00763*	−*2.479*	*0.00562*	−1.895	0.052621	−*3.998*	*0.00731*	Intronic	
	109,392,031	G	A	−0.981	0.20394	0.441	0.124	0.88413	−*3.119*	*0.03766*	−*1.661*	*0.02738*	−0.156	0.851485	−*3.224*	*0.00893*	Intronic	
	109,395,030	G	T	−1.257	0.10273	0.439	−0.153	0.85662	−*3.502*	*0.01850*	−*1.835*	*0.01455*	−0.361	0.665254	−*3.551*	*0.00392*	Intronic	
	109,395,667	G	A	−1.257	0.10273	0.439	−0.153	0.85662	−*3.502*	*0.01850*	−*1.835*	*0.01455*	−0.361	0.665254	−*3.551*	0.00392	Intronic	
	109,395,759	C	T	−1.257	0.10273	0.439	−0.153	0.85662	−*3.502*	*0.01850*	−*1.835*	*0.01455*	−0.361	0.665254	−*3.551*	*0.00392*	Intronic	
	**109,399,707**	T	C	4.252	0.34700	0.002	*33.845*	*0.00067*	−2.773	0.62693							Intronic	
	109,402,641	A	G	4.252	0.34700	0.002	*33.845*	*0.00067*	−2.773	0.62693							Intronic	GH02J109402 (3TFs)
	109,403,362	C	T	−*2.695*	*0.00924*	0.137	−2.134	0.07233	−3.495	0.05588	−*2.786*	*0.00646*	−*2.838*	*0.01112*	−*3.441*	*0.04295*	Intronic	GH02J109402 (3TFs)
	**109,449,440**	**T**	**C**	***24.561***	***0.00462***	**0.002**	***25.264***	***0.00101***	**NA**	**NA**							**Exonic**	**GH02J109448 (6TFs)**
	109,486,830	A	G	10.535	0.08157	0.002	*22.889*	0.00236	5.711	0.56084							Intronic	GH02J109486 (4TFs)

Bold: Synonymous and non‐synonymous variants. Yellow highlighted italics: SNPs supporting association with AD phenotypes Green highlight: Separates Regions 1 and 3 from Region 2. Blue: SNP at 10,9312,004 was the original significant SNP identified in the 2015 study. † GRCh38.

#### 3.1.3 Haplotype in Gene Enhancer Regions

Given that these three regions represent primarily noncoding regions, we further examined each variant to see functional relevance (Table 2). Of 15 SNPs that were significantly associated, more than one‐half (12) were located in enhancer regions.

#### 3.1.4 Replication Datasets: Familial LOAD and Sporadic AD

**FIGURE 1:**
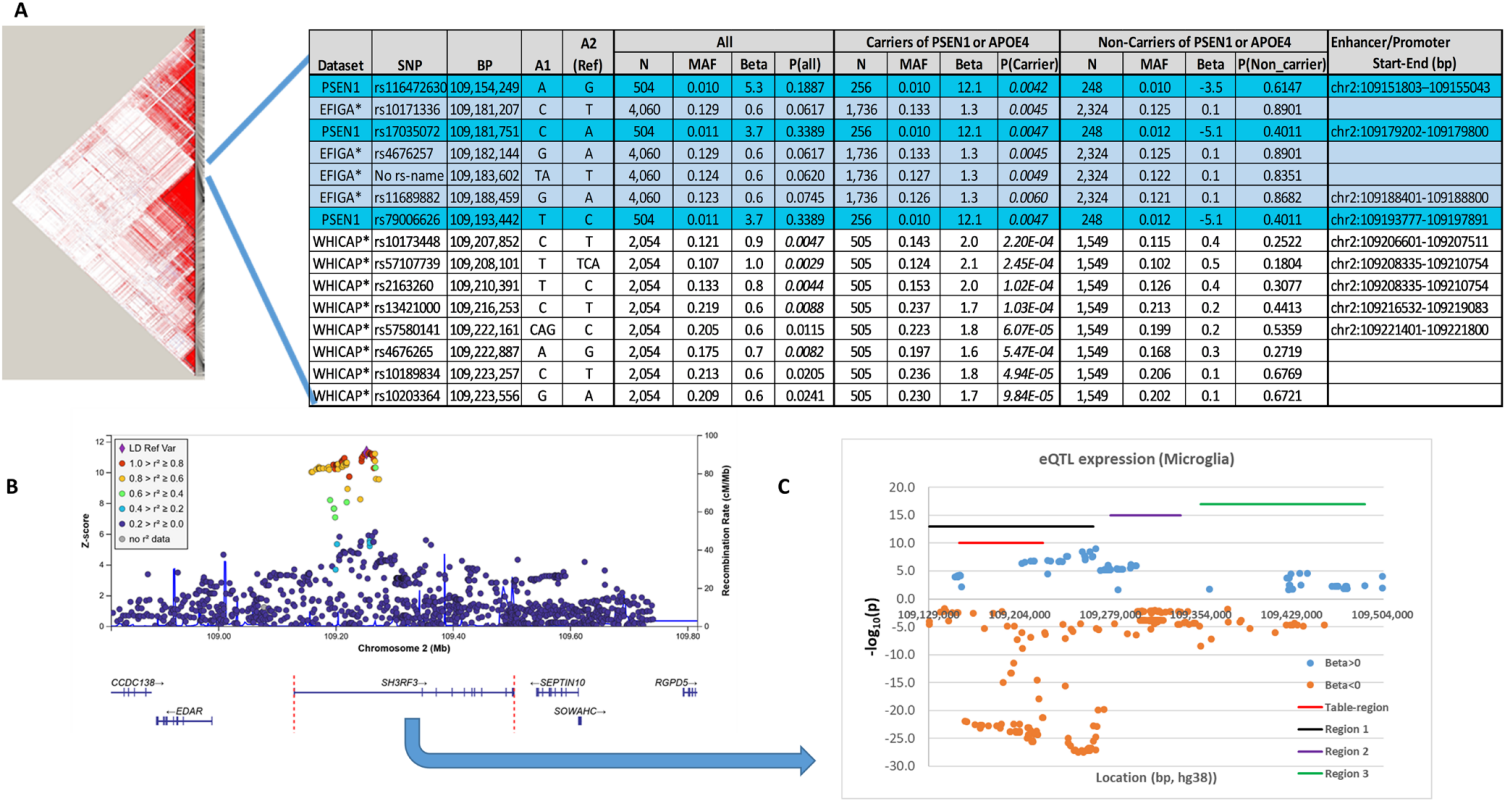
Comparisons of genetic association in *SH3RF3* by cohorts: *PSEN1* ^G206A^, EFIGA, and WHICAP and differential gene expression patterns. (A) Identification of SNPs associated with delay in age at onset of AD in *APOE4* carriers in EFIGA and WHICAP studies. (B) eQTL results for *SH3RF3* and flanking regions, limited to microglia. (C) Directional eQTL expression levels per each locus in *SH3RF3*, where orange represents down‐regulation and blue up‐regulation.

We then examined two other datasets, namely two cohorts of Estudio Familiar de Influencia Genetica en Alzheimer (EFIGA) (Lee et al. 2004) and late onset sporadic AD (WHICAP (Tang et al. 1998)) to determine whether the observed delay in age at onset can be confirmed in independent datasets, and then be generalized to individuals without an autosomal dominant mutation, *PSEN1* ^G206A^. Since these familial LOAD and sporadic AD cases do not carry the *PSEN1* ^G206A^ mutation, we used *APOE4* as the risk variant instead. In these two lower risk cohorts (Figure 1A; Table S1), SNPs in *SH3RF3* were consistently associated with delay in age at onset in *APOE4 carriers*, but the magnitude of delay in age at onset was smaller (range: 1.3–2.9 years) than that in the *PSEN1* ^G206A^ carriers. As in the *PSEN1* ^G206A^ carrier families, allelic associations were not significant in non‐carriers of *APOE4*. This parallels our earlier work in *PSEN1* ^G206A^ mutation carriers, suggesting that genetic modifiers identified from *PSEN1* ^G206A^ mutation carriers can be generalized to other forms of AD. We further note that these variants are located within gene enhancer regions, suggesting that they may influence the regulation of gene expression.

#### 3.1.5 Differential Gene Expression in LOAD

We used the SingleBrain website (https://singlebrain.nygenome.org), a publicly available data portal that provides results from a meta‐analysis of the single nucleus eQTL analysis based on 1047 brain samples from 983 individuals (Jang et al. 2026). This “SingleBrain” webportal combined the samples from four studies, including the ROS/MAP‐Columbia, ROS/MAP‐MIT, Roche, and Seattle Alzheimer's Disease Brain Cell Atlas (SEA‐AD), and provides data on eQTL analysis, colocalization and Mendelian randomization. Figure 1B,C shows the eQTL results in microglia for the SNPs in *SH3RF3*. Each dot represents the log_10_(*p*) for a given SNP. To represent whether a rare allele in a given SNP is down‐ or up‐regulated, we used a two‐color scheme to represent the direction: orange dots represent down regulation, and blue dots represent up regulation. As shown, most eQTLs show down regulation in *SH3RF3* in enhancer regions identified in Table 1. Interestingly, SNPs in these regions had the largest effects in microglia as compared to other CNS cell types (data not shown). In addition, we examined differential gene expression based on bulk RNA sequencing of temporal cortex sample (Table S2). *SH3RF3* was downregulated both in carriers and noncarriers of the *PSEN1* ^*G206A*^ mutation. However, the effect size of the betas was much greater in carriers than in non‐carriers (−0.386 vs. −0.144, respectively), but the difference was not statistically significant.

### 3.2 *SH3RF3* Knockdown Does Not Affect APP Processing in Human Neurons

**FIGURE 2:**
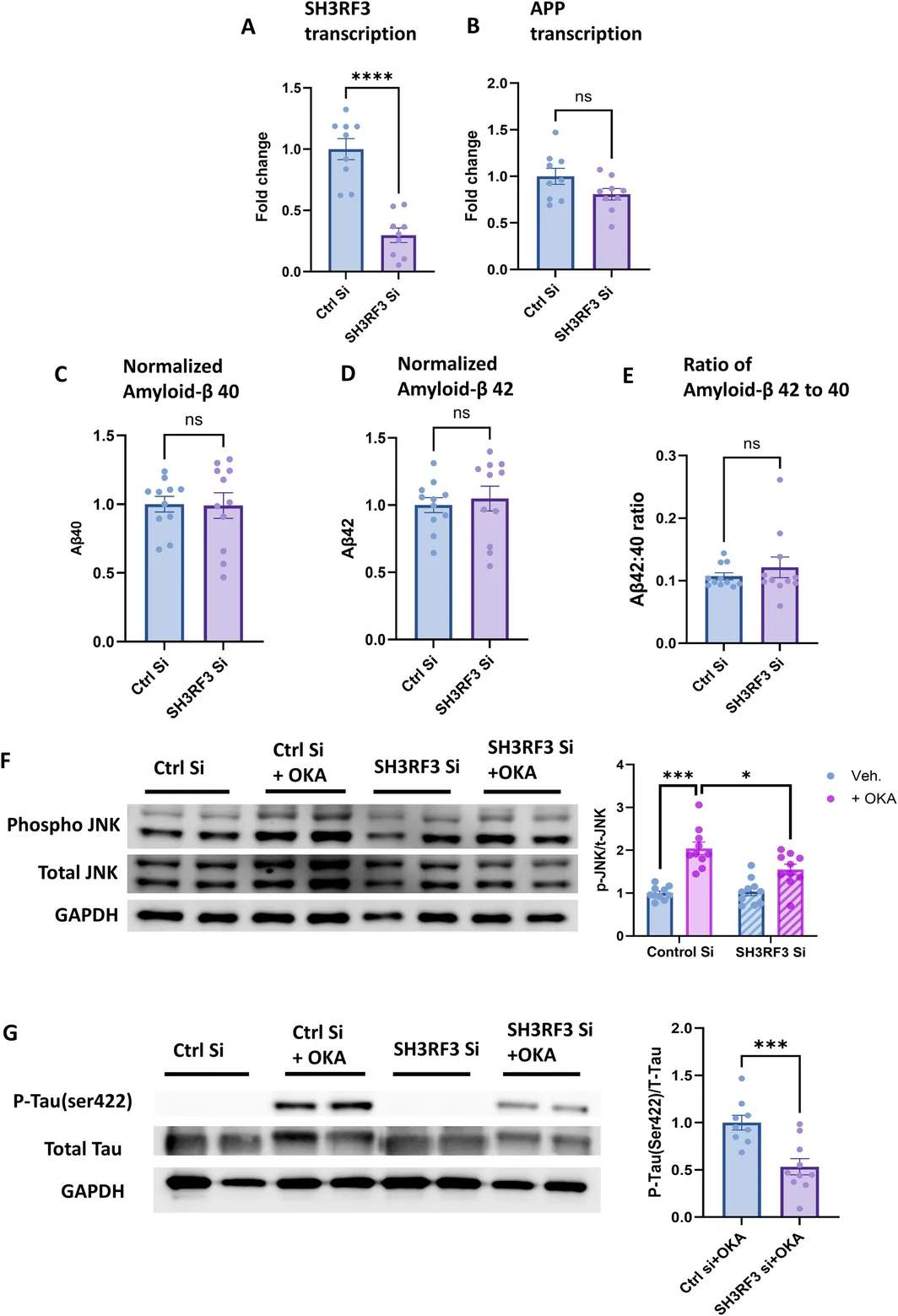
*SH3RF3* KD does not affect APP processing but does reduce pTau S422. (A, B) qPCR analysis of *SH3RF3* and *APP* in hPSC‐derived neurons treated with non‐targeting control or *SH3RF3* siRNA. *SH3RF3* is significantly knocked down but subsequent effects on *APP* are only a trend. Data are presented as mean ± SEM with *N* = 3 biological replicates (independent treated wells) for three independent experiments (independent differentiations). Comparisons are made using an unpaired *t*‐test. (C–E) Multiplex ELISA for Aβ from conditioned media (CM) collected from neurons treated with either *SH3RF3* or control siRNA. Normalized total Aβ42 and total Aβ40 levels are similar for each condition, as is Aβ42/Aβ40 ratio (E). Data are presented as mean ± SEM with *N* = 2–3 biological replicates per experiment for four independent experiments (*N* = 11). Comparisons are made with unpaired *t*‐test. (F, G) Western blot analysis of neurons treated with *SH3RF3* or control siRNA subjected to okadaic acid (OKA) or vehicle for 3 h. Representative blots and quantification are shown. JNK signaling is strongly activated by OKA as measured by pJNK/JNK ratio (F), which is significantly reduced by *SH3RF3* KD. Data are presented as mean ± SEM with *N* = 2–3 biological replicates per experiment for three independent experiments (*N* = 10). Comparisons are made using a mixed‐effects model with treatment group and differentiation as fixed factors, followed by Tukey's multiple comparisons test to determine *p*‐values for the main effect of treatment group (Control si ± OKA vs. *SH3RF3* si ± OKA), ***< 0.001, *< 0.05. pTau S422 phosphorylation (relative to total tau) is strongly induced by OKA treatment (G), which is decreased by *SH3RF3* KD. Data are presented as mean ± SEM with *N* = 2 or 3 biological replicates per experiment from 3 independent experiments (*N* = 7–8). Conditions without OKA have undetectable bands for pTau S422 and only conditions with OKA are quantified by comparison of control si with OKA and *SH3RF3* si with OKA via an unpaired *t*‐test, **< 0.01.

We next sought to understand the mechanisms of how protective SNPs in *SH3RF3* affected AD risk using human model systems. We first assessed loss of *SH3RF3* function in neurons produced by NGN2‐mediated transdifferentiation of hPSCs (H9 hESC line) into excitatory neurons using a permanent line method developed by our lab (Song et al. 2021). We used pooled siRNAs to knockdown (KD) *SH3RF3* in neurons which was verified by qPCR (Figure 2A). We then tested for potential JNK pathway‐related modulation of APP and Aβ production that might be modulated by *SH3RF3* KD. In addition to the effects on APP processing described above for JNK signaling, *APOE* isoforms can differentially activate the AP1 transcription factor to increase *APP* transcription and Aβ production (Huang et al. 2017). The downstream JNK target c‐Jun can also activate AP1 when phosphorylated and dimerized with FOS. *SH3RF3* KD resulted only in a trend of reduced *APP* transcription and total Aβ production (Figure 2B–D; Figure S1A,B). Importantly, the Aβ42:Aβ40 ratio also remained unchanged (Figure 2E). This suggests that while protective SNPs in *SH3RF3* may have subtle beneficial effects on modulating *APP* transcript levels, overall, it is unlikely that their protection against AD is primarily mediated through effects on Aβ production.

### 3.3 *SH3RF3* Knockdown Decreases JNK‐Specific Tau Phosphorylation at ser422

We then assessed effects of *SH3RF3* KD on tau phosphorylation. We first confirmed that *SH3RF3* KD modulates JNK pathway activity in human neurons under basal and JNK pathway‐inducing conditions. The protein phosphatase 2A inhibitor okadaic acid (OKA) activates JNK signaling in cultured hippocampal neurons and induces hyperphosphorylated tau and neuronal cell death (Vogel et al. 2009; Kim et al. 1999). OKA induced activation of JNK signaling in human neurons, which was reduced by *SH3RF3* KD (Figure 2F). S422 has been identified as a prominent JNK‐induced phosphorylation site in tau by mass spectrometry (Reynolds et al. 1997). Tau S422 is expressed at very low levels under physiological conditions and co‐localizes with PHFs in AD brains (Hasegawa et al. 1996), and correlates with tangle formation in murine models of frontotemporal dementia (FTD) and increased tau aggregation (Deters et al. 2008; Haase et al. 2004). While pTau S422 was not detectable under basal conditions, *SH3RF3* KD under OKA treatment conditions demonstrated a reduction in pTau S422 (Figure 2G). On the other hand, there was only a non‐significant trend of decreased pTau T217, a potential blood marker of AD (Montoliu‐Gaya et al. 2023; Barthelemy et al. 2019), from *SH3RF3* KD under OKA treatment conditions (Figure S1C). This suggests that protective SNPs that reduce *SH3RF3* expression levels or function may modulate AD risk in part by affecting levels of phosphorylation of tau at specific phospho sites.

### 3.4 *SH3RF3* KD Reduces Inflammatory Cytokines Produced by iMGLs Treated With the Viral Mimic Poly(I:C) by Modulating JNK and NFκB Signaling

We then tested if *SH3RF3* KD had a more robust effect in microglia, where JNK and NFκB signaling have critical roles under pro‐inflammatory conditions (Merighi et al. 2022; Mehan et al. 2011). Further support from this approach came from looking at interactive published data sets of human and mouse CNS cell types, which showed that in the human brain *SH3RF3* had the highest expression in microglia and fetal astrocytes (Figure S2A; brainrnaseq.org) (Zhang et al. 2016). Interestingly, this was not the case in the mouse brain, where expression was highest in neurons and low in microglia (Figure S2B) (Zhang et al. 2014), suggesting species‐specific differences in *SH3RF3*'s functional role.

Multiple neurodegenerative diseases have shown strong associations with viral exposure. This includes AD, which is correlated with viral encephalitis, herpes simplex infections, HHV6, picornaviruses, and HIV type 1 (Levine et al. 2023; Steel and Eslick 2015). Viral infections are postulated to increase Aβ production as a protective response leading to increased amyloid deposition and induction of inflammatory molecular pathways in the CNS (Liu et al. 2023). This idea is supported by recent findings that show an increase of Aβ in brains from subjects infected by COVID‐19 (Priemer et al. 2022). Various Pathogen Associated Molecular Patterns (PAMPs) can activate Toll‐Like Receptors (TLRs) on the surface of microglia leading to microglial activation and release of inflammatory cytokines in response to microbes like viruses and/or Aβ accumulation, which in turn cause neurotoxic damage (Kumar 2019). Increased release of pro‐inflammatory cytokines is also associated with damage to oligodendrocytes and BBB leakage, leading to exacerbation of AD pathology (Wang et al. 2015; Oligodendrocytes 2026). Inhibitors blocking microglial activation and release of inflammatory cytokines have been proposed as a potential AD therapeutic strategy (Cai et al. 2022).

**FIGURE 3:**
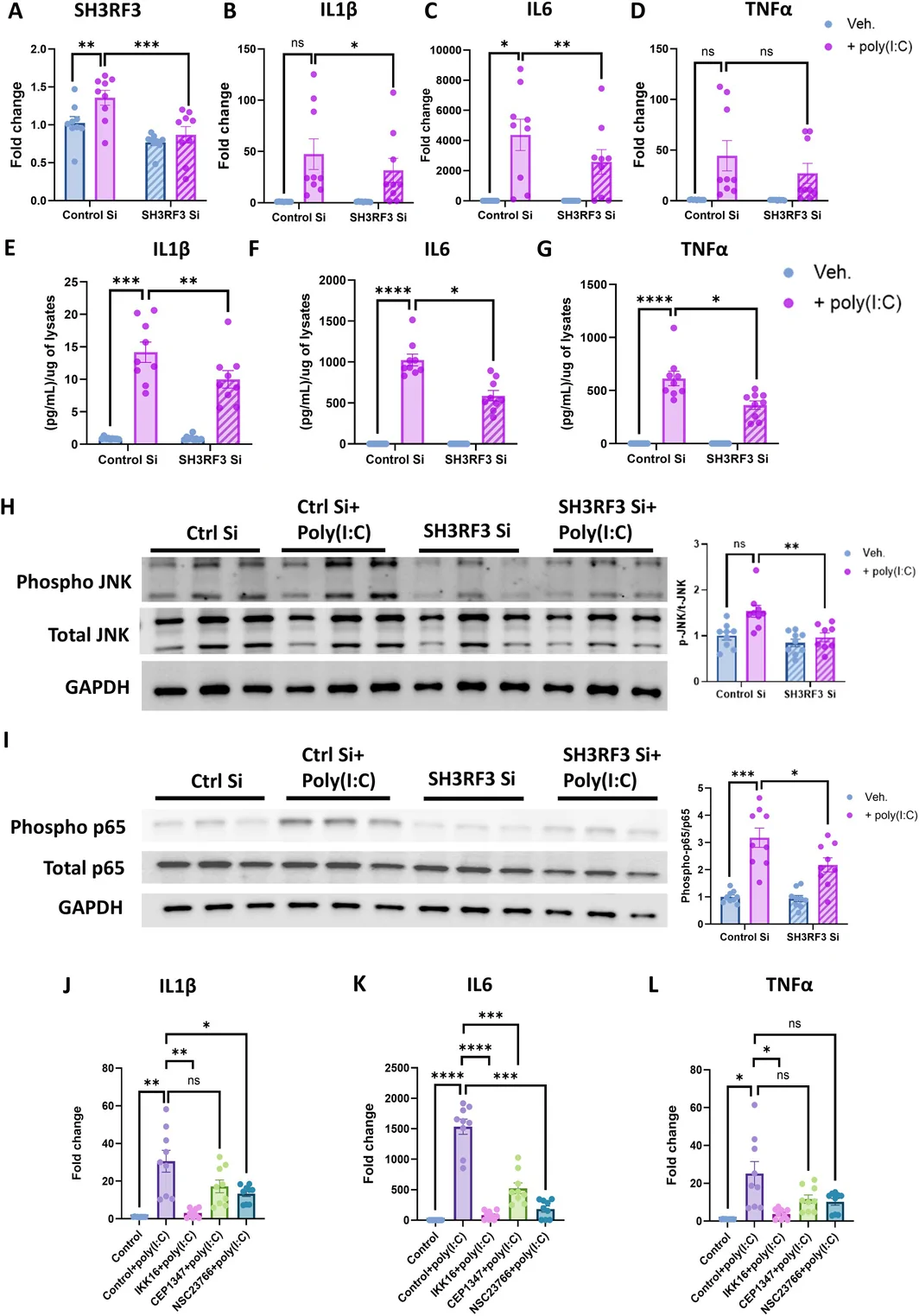
*SH3RF3* knockdown reduces inflammatory cytokine production in iMGLs treated with poly(I:C) via modulation of JNK and NFκB pathways. (A–D) iMGLs treated with either *SH3RF3* or control siRNA were subjected to poly(I:C) or vehicle for 2 h. qPCR analysis of *SH3RF3* (A), *IL1β* (B), *IL6* (C), and *TNFα* (D) showed successful knockdown of *SH3RF3* and induction of all cytokines upon poly(I:C) treatment which were significantly reduced by *SH3RF3* KD. Data are presented as mean ± SEM for *N* = 3 biological replicates (independent wells) per experiment for three independent experiments (independent differentiations; *N* = 9). Comparisons are made using a mixed‐effects model with treatment group and differentiation as fixed factors, followed by Tukey's multiple comparisons test to determine *p*‐values for the main effect of treatment group (Control si ± poly(I:C) vs. *SH3RF3* si ± poly(I:C)), ***< 0.001, **< 0.01, *< 0.05, ns = not significant. (E–G) Multiplex ELISA analysis of inflammatory cytokines from conditioned media from iMGLs treated with poly(I:C) for 16 h. IL1β (E), IL6 (F), and TNFα (G) all show significant induction by poly(I:C) which is reduced by *SH3RF3* KD. Data are presented as mean ± SEM for *N* = 3 biological replicates per experiment for three independent experiments (*N* = 9). Comparisons are made using a mixed‐effects model with treatment group and differentiation as fixed factors, followed by Tukey's multiple comparisons test to determine *p*‐values for the main effect of treatment group (Control si ± poly(I:C) vs. *SH3RF3* si ± poly(I:C)), ****< 0.0001, ***< 0.001, **< 0.01, *< 0.05. (H–I) Western blot analysis of JNK and NFκB pathway activation from iMGLs treated with *SH3RF3* or control siRNA subjected to poly(I:C) for 6 h. Representative blots for phosphorylated JNK and total JNK (H), phosphorylated p65 and total p65 (I) with densitometry quantification for 3 independent experiments. GAPDH is used as loading control. Data are presented as mean ± SEM for *N* = 3 biological replicates per experiment for three independent experiments (*N* = 9). Comparisons are made using a mixed‐effects model with treatment group and differentiation as fixed factors, followed by Tukey's multiple comparisons test to determine *p*‐values for the main effect of treatment group (Control si ± poly(I:C) vs. *SH3RF3* si ± poly(I:C)), ***< 0.001, **< 0.01, *< 0.05, ns = not significant. (J–L) qPCR analysis of *IL1β* (J), *IL6* (K), and *TNFα* (L) in iMGLs treated with poly(I:C) for 2 h, with the presence or absence of pathway inhibitors, all of which reduce transcription of all 3 cytokines. CEP‐1347 is a JNK pathway (MLK/MAP3K) inhibitor, IKK16 is a NFκB pathway inhibitor, and NSC23766 is a Rac1 inhibitor. Data are presented as mean ± SEM for *N* = 3 biological replicates per experiment for three independent experiments (*N* = 9). Comparisons are made using a mixed‐effects model with treatment group and differentiation as fixed factors, followed by Tukey's multiple comparisons test to determine *p*‐values for the main effect of treatment group (Control ± poly(I:C) vs. IKK16 or CEP‐1347 or NSC23766 ± poly(I:C)), ****< 0.0001, ***< 0.001, **< 0.01, *< 0.05, ns = not significant.

Thus, we utilized a viral mimic poly(I:C) to treat iMGLs, double‐stranded RNA (dsRNA) known to elicit inflammatory microglial response via TLR3 activation (He et al. 2021). iMGLs were differentiated from the FA0000010 (FA10) hiPSC line using a previously published protocol with minor modifications (McQuade et al. 2018). Most differentiated cells were CD45^+^/CD11B^+^ as measured by flow cytometry (Figure S2C) and expressed key microglial markers such as Iba1 and P2RY12 by immunostaining (Figure S2D). We used similar pooled non‐targeting control siRNA and siRNA targeting *SH3RF3* in iMGLs to knockdown *SH3RF3* in the presence and absence of poly(I:C) stimulation. We observed moderate knockdown of *SH3RF3* by qPCR (Figure 3A). For assessing effects on pro‐inflammatory cytokine production, we focused on 3 key inflammatory cytokines, *IL1β*, *IL6* and *TNFα*, which are also members of the human‐specific CRM2 microglial cluster that appears in response to amyloid (Mancuso et al. 2024). *IL1β*, *IL6*, and *TNFα* transcript levels upon 2 h poly(I:C) stimulation showed upregulation compared to vehicle treated iMGLs, of which *IL1β* and *IL6* were significantly reduced by *SH3RF3* KD in poly(I:C) conditions, and a small increase in *SH3RF3* transcript was seen from poly(I:C) treatment (Figure 3A–D). This also translated to the protein level as measured by multiplex ELISA for IL1β, IL6, and TNFα from conditioned media from iMGLs at 16 h post‐treatment. Poly(I:C) treatment resulted in significantly higher release of IL1β, IL6, and TNFα which was significantly reduced by *SH3RF3* KD for all 3 cytokines (Figure 3E–G).

We then investigated whether modulation of JNK and NFκB signaling pathways was responsible for the *SH3RF3* KD reduced inflammatory cytokine phenotype in iMGLs. *SH3RF3* KD was performed in presence and absence of poly(I:C) treatment for 6 h and lysates were subjected to western blot. We observed increased JNK phosphorylation (Thr183 and Tyr185), and hence increased JNK activity, for multiple JNK isoforms upon poly(I:C) treatment (Figure 3H), albeit only a trend when 3 independent experiments data were combined. *SH3RF3* KD showed reduced JNK phosphorylation compared to control siRNA poly(I:C) treated conditions. We also assessed if poly(I:C) treatment of iMGLs could induce activation of the p65 (Rel‐A) subunit of NF‐kB signaling and the effect of *SH3RF3* KD on NF‐kB activation. We observed significantly upregulated p65 phosphorylation, demonstrating strong activation of NF‐kB signaling, which also was reduced by *SH3RF3* KD. (Figure 3I). Similar results were seen for the p105 subunit NFKB1 (Figure S2E). In summary, *SH3RF3* levels regulate activity of JNK and NF‐kB signaling consistent with these pathways mediating inflammatory response in iMGLs upon poly(I:C) stimulation.

### 3.5 Pharmacological Inhibitors of JNK and NF‐kB Phenocopy *SH3RF3* KD

To further solidify the mechanistic link between reduced JNK and NF‐kB signaling and reduced cytokine production in response to poly(I:C) treatment, we sought to determine if the effects of *SH3RF3* KD on inflammatory cytokine production could be mimicked by pharmacological inhibition of these pathways. To inhibit JNK signaling, we employed CEP‐1347, a semisynthetic mixed lineage kinase (MLK) inhibitor, which blocks JNK activation by inhibiting upstream MLK family member MAP3Ks (Maroney et al. 2001). CEP‐1347 was used in a clinical trial for Parkinson's disease, where it failed due to efficacy rather than safety concerns (Parkinson Study Group, P.I 2007). We also used IKK16, a selective inhibitor of IkB kinase resulting in inhibition of canonical NF‐kB signaling (Waelchli et al. 2006). We treated iMGLs with poly(I:C) in the presence of CEP‐1347, IKK16, or DMSO vehicle control and assessed inflammatory cytokine transcription. IKK16 produced a significant reduction in the transcription of all three inflammatory cytokines, whereas CEP‐1347 only had statistical effects on *IL1β* and *IL6* (Figure 3J–L). *SH3RF3* interacts with activated RAC1 upstream to JNK and NF‐kB kinases (Zhang et al. 2009), which led us to hypothesize that inhibition of RAC1 activity might act similarly to *SH3RF3* KD (Karkkainen et al. 2010). Treatment with the RAC inhibitor NSC23766 also reduced IL1β and IL6 production in poly(I:C) stimulated iMGLs, although it was used at a high concentration (50 μM) due to its low potency (Figure 3J–L) (Discovery 2026). Taken together, this data demonstrates that inhibition of JNK or NF‐kB signaling is sufficient to lower inflammatory cytokine production in iMGLs.

### 3.6 *SH3RF3* KD Reduces Inflammatory Cytokines Produced by iMGLs Treated With Oligomeric Aβ42 by Modulating JNK and NF‐kB Signaling

**FIGURE 4:**
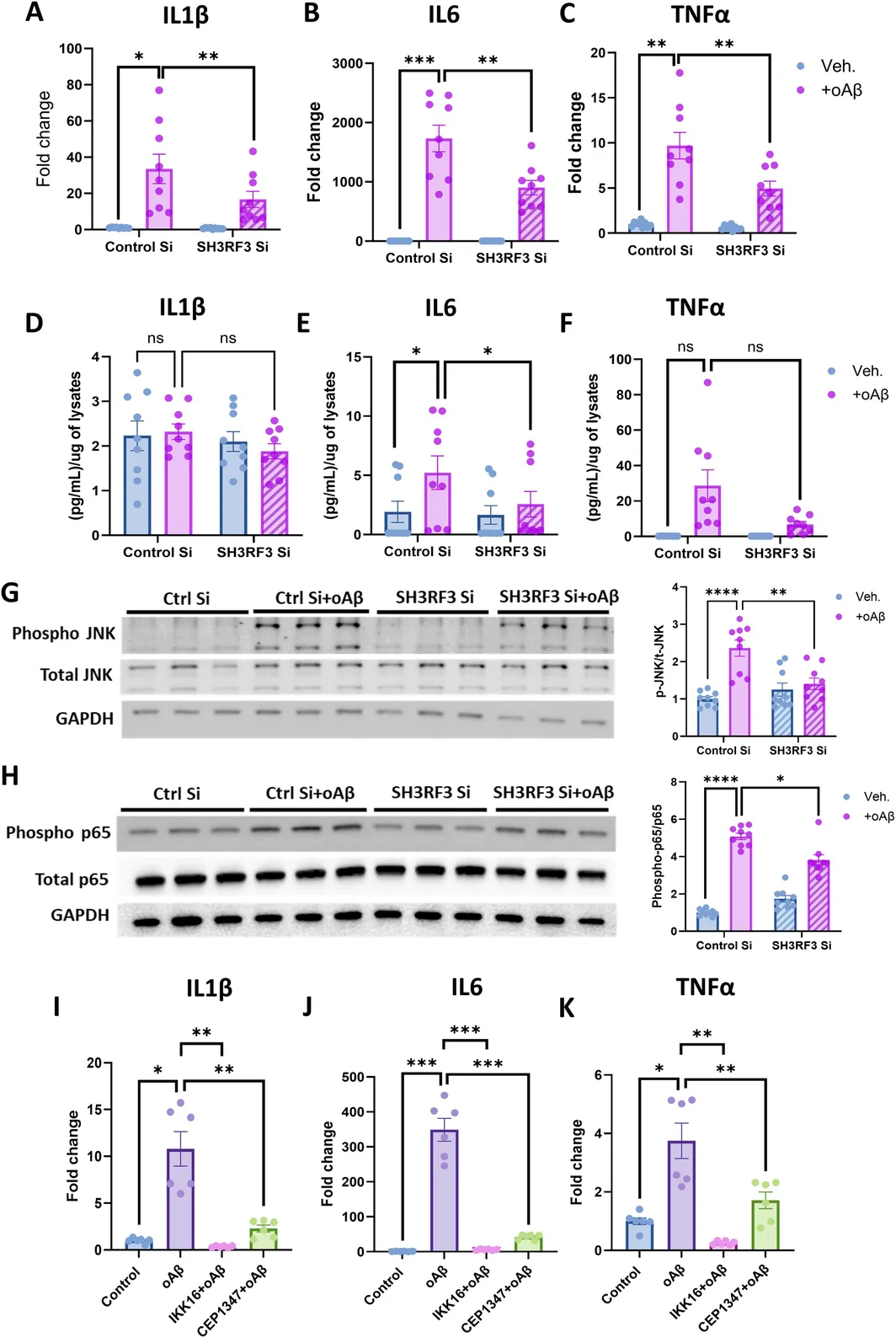
*SH3RF3* knockdown reduces inflammatory cytokines and JNK/NFκB signaling in iMGLs treated with oAβ42. (A–C) qPCR analysis of inflammatory cytokines *IL1β* (B), *IL6* (C), and *TNFα* (D) in iMGLs treated with *SH3RF3* or control siRNA subjected to 5 μM oAβ42 or vehicle for 24 h. Inflammatory cytokines were statistically reduced by *SH3RF3* KD, data are presented as mean ± SEM for *N* = 3 biological replicates (independent wells) per experiment for three independent experiments (independent differentiations; *N* = 9). Comparisons are made using a mixed‐effects model with treatment group and differentiation as fixed factors, followed by Tukey's multiple comparisons test to determine *p*‐values for the main effect of treatment group (Control si ± oAβ42 vs. *SH3RF3* si ± oAβ42), ***< 0.001, **< 0.01, *< 0.05. (D–F) Multiplex ELISA analysis of inflammatory cytokines IL1β, IL6 (E), and TNFα (F) from conditioned media from iMGLs treated with *SH3RF3* or control siRNA subjected to 5 μM oAβ42 for 3 h. TNFα is statically reduced by *SH3RF3* KD. Data are presented as mean ± SEM for *N* = 3 biological replicates per experiment for three independent experiments (*N* = 9). Comparisons are made using a mixed‐effects model with treatment group and differentiation as fixed factors, followed by Tukey's multiple comparisons test to determine *p*‐values for the main effect of treatment group (Control si ± oAβ42 vs. *SH3RF3* si ± oAβ42), **< 0.01, ns = not significant. (G, H) Representative immunoblots and densitometry analysis of JNK (pJNK/JNK, G) and NFkB (phosho‐p65/p65, H) pathway activation in iMGLs treated with *SH3RF3* or control siRNA subjected to oAβ42 or vehicle for 3 h. Densitometry data are presented as mean ± SEM for *N* = 3 biological per experiment for three independent experiments (*N* = 9). Comparisons are made using a mixed‐effects model with treatment group and differentiation as fixed factors, followed by Tukey's multiple comparisons test to determine *p*‐values for the main effect of treatment group (Control si ± oAβ42 vs. *SH3RF3* si ± oAβ42), ****< 0.0001, **< 0.01, *< 0.05. (I–K) qPCR analysis of inflammatory cytokines *IL1β* (I), *IL6* (J), and *TNFα* (K) upon 5 μM oAβ42 treatment for 12 h in iMGLs pre‐treated with either DMSO vehicle, CEP‐1347 (JNKi), or IKK16 (NFκBi). Data are presented as mean ± SEM for *N* = 3 per experiment for two independent experiments (*N* = 6). Comparisons are made using a mixed‐effects model with treatment group and differentiation as fixed factors, followed by Tukey's multiple comparisons test to determine *p*‐values for the main effect of treatment group (Control ± oAβ42 vs. IKK16 or CEP‐1347 or NSC23766 ± oAβ42), ***< 0.001, **< 0.01, *< 0.05.

We next wanted to extend our findings regarding effects of *SH3RF3* KD on cytokine production to another AD‐relevant inflammatory stimuli. Oligomeric amyloid‐β (oAβ) peptides are key drivers of AD according to the amyloid hypothesis (Selkoe and Hardy 2016) and cause microglial activation and release of inflammatory cytokines (Dhawan et al. 2012; Parajuli et al. 2013). We utilized a commonly used Aβ oligomerization protocol (Fa et al. 2010) which we then assessed for oligomerization state. Western blot using the 6E10 Aβ antibody showed the presence of both trimers and higher‐order oligomers (Figure S3A), oAβ42 treatment of iMGLs for 24 h resulted in induction of inflammatory cytokines as assessed by qPCR for *IL1β*, *IL6*, and *TNFα* transcripts compared to vehicle‐treated controls (Figure 4A–C). Corroborating with our previous results with poly(I:C) stimulated iMGLs, *SH3RF3* KD produced significantly reduced transcripts for all 3 cytokines.

Although we initially started with 24 h treatment as a point of analysis, we realized that oAβ42 would also be actively phagocytosed by iMGLs which would reduce the potency of the stimulus over time. We hypothesized this could at least partially explain a recently published study which only showed minor transcriptional effects of iMGLs treated for 24 h with oAβ42 (Quiroga et al. 2022), although that study also used a lower concentration of oAβ42 (1 μM instead of 5 μM) and potentially had a different Aβ oligomerization state. Thus, we did a time course experiment, which showed transcriptional responses at 24 h to oAβ42 were already much lower than at earlier 6 and 12 h time points (Figure S3B). We also determined that NF‐kB signaling was activated by 3 h as assessed by western blot and was more robust for 5 μM as compared to 1 μM oAβ42 (Figure S3C).

Upon learning that oAβ42 transcriptional effects were blunted by 24 h post‐treatment, we tested if iMGLs treated with oAβ42 for 3 h released inflammatory cytokines and if *SH3RF3* KD affected this response. While IL1β and TNFα levels were not increased by oAβ42 at this time point, IL6 levels were significantly elevated (Figure 4D–F). *SH3RF3* KD significantly reduced IL‐6 cytokine production in response to oAβ42. We also evaluated effects of oAβ42 on JNK and NF‐kB pathways in the presence of *SH3RF3* KD. As anticipated, oAβ42 activated JNK and NF‐kB pathways as observed by increased phosphorylation of JNK and p65 (Figure 4G,H) as well as p105 (Figure S3D). Similar to poly(I:C) treatment, *SH3RF3* KD reduced activation of JNK and NF‐kB signaling. We also performed similar chemical inhibitor experiments which demonstrated that JNK inhibition by CEP‐1347 or NF‐kB by IKK16, were sufficient to block inflammatory cytokine production by oAβ42 in iMGLs (Figure 4I–K). In summary, inflammatory cytokines produced by iMGLs treated with oAβ42 are also attenuated by *SH3RF3* KD, which is likely mediated by reduced activation of JNK and NF‐kB signaling.

### 3.7 *PSEN1* ^G206A^ Microglia Have Reduced Induction of Cytokines in Response to Oligomeric Aβ42

**FIGURE 5:**
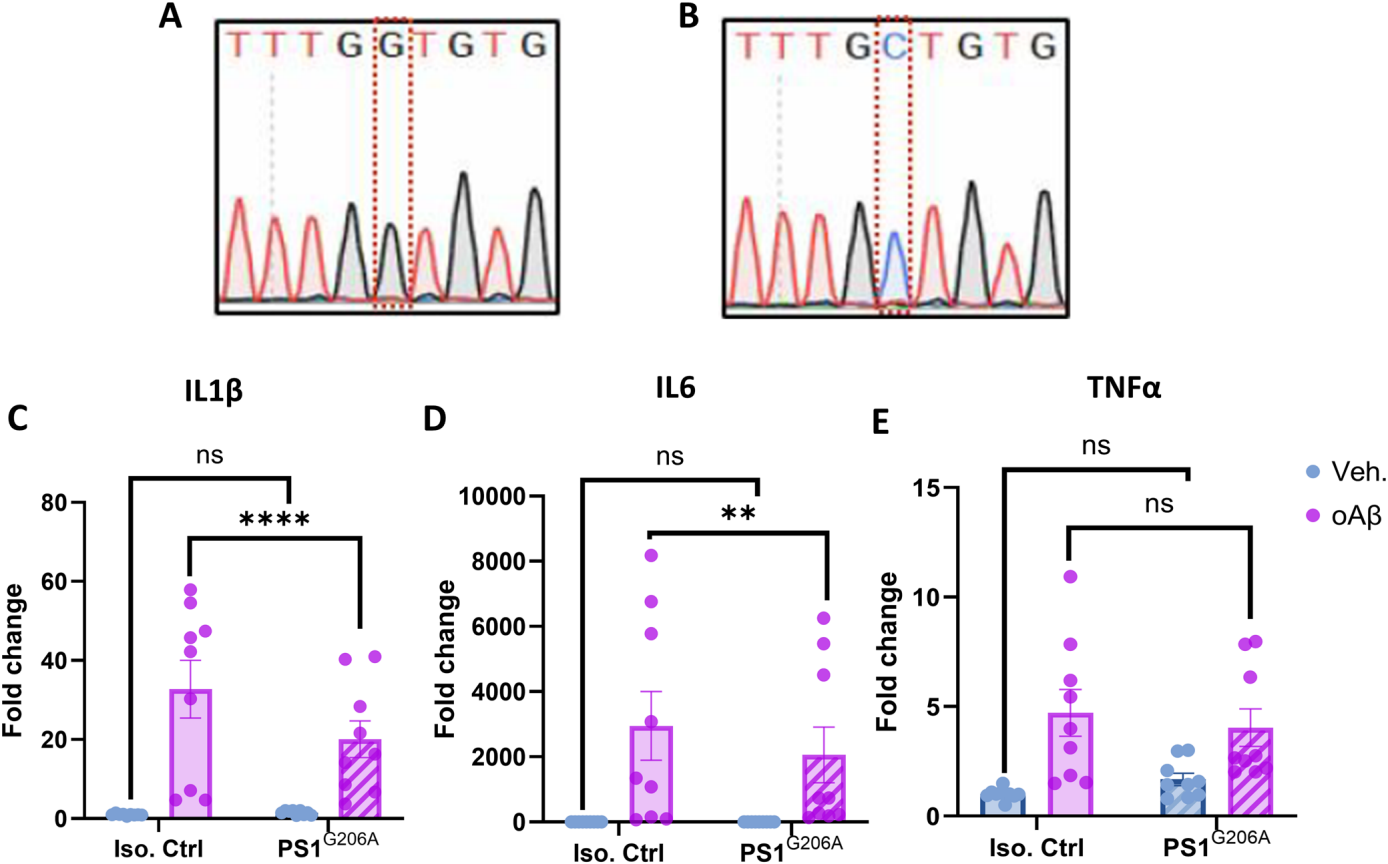
*PSEN1* ^G206A^ microglia have reduced inflammatory cytokine production in response to oAβ42. (A, B) Chromatographs showing (A) isogenic control and (B) *PSEN1* ^G206A^ homozygous knock‐in, as shown by G instead of C (GRCh38) Chr14:73192712G>C. (C–E) qPCR analysis of inflammatory cytokines *IL1β* (C), *IL6* (D), and *TNFα* (E) upon vehicle or 5 μM oAβ42 treatment for 6 h in isogenic control and *PSEN1* ^G206A^ iMGLs. Data are presented as mean ± SEM for *N* = 3 biological replicates (independent wells) per experiment for three independent experiments (independent differentiation; *N* = 9). Comparisons are made using a mixed‐effects model with treatment/genotype group and differentiation as fixed factors, followed by Tukey's multiple comparisons test to determine *p*‐values for the main effect of treatment group (Iso ctrl ± oAβ42 vs. *PSEN1* ^G206A^ ± oAβ42), ****< 0.0001, **< 0.001, ns = not significant.

We hypothesized that *SH3RF3* protective SNPs might be particularly beneficial in *PSEN1* mutation carriers due to their strong effect on age of onset in that *PSEN1* ^G206A^ (Table 2). It has been previously reported that *PSEN1* ^ΔE9^ iMGLs have reduced inflammatory responses to bacterial *lipopolysaccharide* (LPS) relative to isogenic control iMGLs (Konttinen et al. 2019). To test if *PSEN* ^G206A^ iMGLs also have reduced inflammatory responses, we knocked the G206A mutation into both alleles of *PSEN1* in the IMR90 (cl.4) hiPSC line using CRISPR/Cas9 (Figure 5A,B). Homozygous rather than hemizygous knock‐in was confirmed via qgPCR (data not shown) (Weisheit et al. 2020). While biallelic knock‐in of the G206A mutation would be expected to have stronger phenotypes than a heterozygous knock‐in while maintaining physiological expression, it is important to note that almost all mutation carriers would only have one copy in human subjects. *PSEN1* ^G206A^ and isogenic control iMGLs were then treated with oAβ42 for 6 h to look for transcriptional effects on *IL1β*, *IL6*, and *TNFα* (Figure 5C–E; Figure S4). While the level of transcriptional response to oAβ42 was more variable between independent experiments using these lines, *IL1β* and *IL6* were consistently reduced in *PSEN1* ^G206A^ iMGLs compared to isogenic control iMGLs treated with oAβ42. Overall, this suggests that microglia in subjects with *PSEN1* mutations may have reduced pro‐inflammatory responses in general.

## 4 Discussion

It is critical to understand the mechanisms of why some high‐risk individuals are protected from getting AD as an alternative therapeutic strategy to targeting neuropathological changes in amyloid and tau directly. In this study, we focused on *SH3RF3*, which we previously identified as protective against AD in *PSEN1* ^G206A^ carriers (Lee et al. 2015). Our discovery dataset of *PSEN1* ^G206A^ carrier families yielded a set of contiguous SNPs with a varying degree of delay in age at onset. Then the subsequent examination of familial and sporadic LOAD in Caribbean Hispanics (using *APOE4* as a risk variant) confirmed the modifying effects of *SH3RF3*, in which the significant association was observed in the presence of *APOE4*, but not in the absence of *APOE4*. We further note that these variants were located in gene enhancer regions, supporting the observed varying degree of gene expressions.

Thus, we then sought to understand the mechanisms of how a protective SNP in *SH3RF3* (Lee et al. 2015) delayed onset of AD in *PSEN* ^G206A^ carriers. We focused on JNK and NF‐kB signaling since SH3RF family members including *SH3RF3* had been shown to be able to activate these pathways (Karkkainen et al. 2010; Tapon et al. 1998; Zhang et al. 2020; Xu et al. 2003). For JNK signaling, SH3RF proteins (as best described for SH3RF1/POSH) act as scaffolds linking upper and lower components of this MAPK cascade (Xu et al. 2003; Kukekov et al. 2006). The mechanism(s) of how *SH3RF3* modulates NF‐kB activity is not well understood and should be the subject of further research. We initially focused on neurons, as early‐onset AD mutations like those in *PSEN1* are primarily thought to drive disease through modulating APP processing, such as by increased Aβ42:Aβ40 ratio. However, *SH3RF3* KD did not affect APP processing in a significant fashion under steady‐state conditions (Figure 2), although the small decrease in APP transcription (not statistically significant) could have some impact over the long course of human aging. *SH3RF3* KD did decrease phosphorylation at the JNK‐specific tau S422 phospho‐site which could have some effect on AD risk. It is important to note that while phosphorylation of the S422 site of tau is found in PHFs in AD brains (Hasegawa et al. 1996) and has been proposed to be pathogenic (Ma et al. 2009), it also has been reported that phosphorylation of tau at S422 blocks caspase‐3 cleavage of tau (Guillozet‐Bongaarts et al. 2006), which is considered a pro‐pathogenic event. In addition, one significant caveat of our neuronal studies is that as JNK and NF‐kB are stress‐induced signaling cascades (reviewed in (Kim and Choi 2010; Merighi et al. 2022; Kaminska et al. 2009; Liu et al. 2017)), thus, *SH3RF3* may play a stronger role in neurons in response to specific stresses not tested here.

We then chose to look at microglia as JNK and NF‐κB signaling are also critical for inflammatory responses in this cell type (Merighi et al. 2022; Mehan et al. 2011). We chose to focus our analysis on 3 pro‐inflammatory cytokines, *IL1β*, *IL6*, and *TNFα*, which have been associated with AD and are members of a human‐specific microglial cytokine response cluster (CRM2) activated in human microglia xenotransplanted into the humanized APP^NL‐G‐F^ AD mouse model (Mancuso et al. 2024). *SH3RF3* KD can blunt pro‐inflammatory cytokine production from two AD‐relevant stresses, a viral mimic poly(I:C) and oAβ42, chosen in part for association with JNK and NF‐κB signaling (Figures 3, 4, 5). *SH3RF3* KD was associated with reduced JNK and NF‐κB signaling, and blockade of these pathways through chemical inhibitors was sufficient to reduce inflammatory cytokine production. Overall, our data are consistent with a model where reduced expression of *SH3RF3* by protective SNPs lowers inflammatory cytokines produced by inflammatory insults by dampening JNK and NF‐κB signaling, to consequently delay the onset of AD (see *Graphical Abstract*). It is important to note that while poly(I:C) and oAβ42 treatments in iMGLs have some similar aspects in terms of signaling cascades being activated and subsequent upregulation of pro‐inflammatory cytokines, a deeper cross comparison would certainly show distinct effects between these inflammatory stimuli in general and in response to *SH3RF3* KD. In addition, although our data suggests that *SH3RF3* acts through modulating JNK and NF‐κB signaling, it is possible that it has additional functions such as through its E3 ligase activity that could affect inflammatory processes independently of these pathways. Interestingly, while genes expressed primarily in microglia have almost exclusively been associated with late‐onset forms of AD, and early‐onset AD has been associated with neuronal acting genes, our study suggests that early‐onset AD risk may also be significantly influenced by genetic modulators of microglial function. Why microglia from *PSEN1* mutation carriers (ΔE9 (Konttinen et al. 2019) or G206A) (Figure 5) have reduced inflammatory responses remains unclear. As TREM2 is a direct γ‐secretase target (Wunderlich et al. 2013), an intriguing possibility is that *PSEN1* mutant microglia have reduced/altered γ‐secretase activity against TREM2 which leads to enhanced TREM2 intracellular signaling and reduced inflammatory processes, which could be investigated in future studies.

There are several additional limitations to our study, including that we focused exclusively in microglia on pro‐inflammatory cytokine production. We also only focused on 3 relevant cytokines, and a broader cytokine profile would likely show significant differences between poly(I:C) and oAβ42 treatments and would further delineate which of these molecules are dependent on *SH3RF3*. Other inflammatory responses such as ROS production or effects on phagocytosis should be addressed in future studies. Regarding inflammatory reactions of iMGLs to oAβ42, it is important to note that this is a very transient response that could reflect elevated oAβ from transient pathological events like ischemia which increase Aβ levels (Salminen et al. 2017), rather than a more “DAM”‐like response to aggregated fibrillar forms of Aβ (Dolan et al. 2023). On the other hand, xenotransplantation of iMGLs into the APP^NL‐G‐F^ AD mouse model (Mancuso et al. 2024) had human‐specific cytokine modules under both steady‐state responses to amyloid and co‐injection of oAβ42, suggesting a broader role of inflammatory cytokine production in the human microglial response to AD pathology. Importantly, *SH3RF3* was a member of the CRM2 module suggesting it could help drive this cluster which could be tested in future xenotransplantation experiments. It should also be noted that the concentration of oAβ42 used to treat iMGLs (5 μM) is hyperphysiological, and that looking at repeated doses of a lower concentration of oAβ42 in future studies would be more physiological and likely informative.

An important remaining question is how *SH3RF3* is regulated during responses to inflammatory stimuli in microglia. Our data shows only a very small increase in *SH3RF3* transcript in response to poly(I:C) (Figure S2E). This would argue that an increase in transcription is not responsible for *SH3RF3*'s effects on JNK and NFkB signaling during inflammatory reactions or in AD brains. We favor a model in which SH3RF3 protein is stabilized in response to strong inflammatory signaling to promote these signaling cascades, by prevention of self‐targeting for proteasomal degradation via its E3 ligase activity. A similar model has been shown for its homologue *SH3RF1*/*POSH* in response to apoptotic stimuli (Xu et al. 2005). SNPs that affect *SH3RF3* transcript levels would in turn affect the available protein pool to be stabilized during inflammatory (and likely apoptotic) stimulation. A non‐mutually exclusive alternative is that SH3RF3 protein changes its binding partners and subcellular localization during inflammatory stimulation to promote JNK/NFkB signaling cascades, which would also be dependent on the available transcript pool. We attempted to look at endogenous SH3RF3 protein by using multiple commercial antibodies as well as two attempts to make our own chicken antibody to SH3RF3 using a commercial vendor, although none of these antibodies could not reliably detect epitope‐tagged overexpressed *SH3RF3* let alone endogenous protein. We are currently using CRISPR to edit endogenous *SH3RF3* with an epitope tag which will allow us to address *SH3RF3* protein regulation in future studies.

Finally, a key goal of trying to understand the molecular mechanisms of AD protective SNPs was to mimic those genetic protective mechanisms with small molecules. We found CEP‐1347, a MAP3K (inhibitor that blocks JNK signaling (Maroney et al. 2001)), significantly reduced inflammatory cytokine production in iMGLs from both a viral mimic and oAβ42. CEP‐1347 and other MAP3K inhibitors have previously shown benefits in blunting inflammatory responses in other microglial/macrophage models and promote Aβ clearance (Dong et al. 2016; Lund et al. 2005; Kiyota et al. 2018). Importantly, CEP‐1347 has also been shown to block neuronal cell death from oAβ42, trophic factor withdrawal, UV, and oxidative stress (Troy et al. 2001; Maroney et al. 1999). This data led, at least in part, to CEP‐1347 being tested in the PRECEPT trial for Parkinson's disease (PD), where it failed for efficacy rather than safety concerns (Waldmeier et al. 2006). One possible reason for this failure was that neuronal cell death in PD has been reported to be driven by the MAP3K DLK (*dual leucine zipper kinase*) (Chen et al. 2008), which CEP‐1347 inhibits to a much lesser degree than MLKs (Maroney et al. 2001). In addition, genetic evidence suggests that microglia play a more dominant role in AD than PD, and therefore, CEP‐1347 effects on microglial inflammatory responses would be expected to have greater importance in AD. Thus, reconsideration of using MAP3K (MLK) inhibitors like CEP‐1347 to treat AD may be warranted as this could act as a double hit to both dampen pro‐inflammatory microglial responses and to protect neurons from cell death from Aβ and other insults. This particularly could be the case for *PSEN1* mutant carriers, as we (Figure 5F) and others (Konttinen et al. 2019) have seen a lower baseline inflammatory response in *PSEN1* mutant microglia, as well as *PSEN1* mutant carriers having a more clearly Aβ‐driven form of AD.

## Author Contributions

R.P., R.C., C.L.C., G.S., E.T., S.M., and A.A. conducted experiments and analyzed data. A.P., A.M., and I.Z.J.‐V. contributed to the collection and analysis of *PSEN1* and control subject samples. R.M. provided additional data sets for replication of the findings from the initial analysis. R.P., R.C., J.H.L., and A.A.S. designed experiments and wrote the manuscript. All authors edited the manuscript. A.A.S. and J.H.L. supervised all research.

## Funding

This work was primarily supported by NIA R01AG058918 (J.H. PI, AAS Co‐I) and R56 AG051876 (J.H.L.). It was also supported by BrightFocus (A2015633S; J.H.L.), 1P30AG066462‐01 (A.A.S. Co‐PI on Development grant), the Henry and Marilyn Taub Foundation (A.A.S.), and the Thompson Family Foundation (TAME‐AD, A.A.S.). Data used in the replication analyses were supported by R01AG067501 (R.M.) and U24AG056270 (R.M.).

## Conflicts of Interest

The authors declare no conflicts of interest.

## Supporting information


**Figure S1:** *SH3RF3* KD does not significantly affect APP processing and p‐tau T217. (A, B) Multiplex ELISA for Aβ from conditioned media (CM) collected from neurons treated with either *SH3RF3* or control siRNA. Absolute values for Aβ40 and Aβ42 corresponding to Figure 2C,D showing no change in Aβ production. Data are presented as mean ± SEM with *N* = 2–3 biological replicates per experiment for four independent experiments (*N* = 11). Comparisons are made using an unpaired *t*‐test. (C) Western blot analysis of Tau (Thr217) phosphorylation with 3 h OKA treatment with non‐targeting siRNA and *SH3RF3* siRNA in neurons. Representative blots and quantification is presented. Data are presented as mean ± SEM with *N* = 3 from two independent experiments. Comparisons are made using a mixed‐effects model with treatment group and differentiation as fixed factors, followed by Tukey's multiple comparisons test to determine *p*‐values for the main effect of treatment group (Control si ± OKA vs. *SH3RF3* si ± OKA), ***< 0.001, NS.

**Figure S2:** iMGLs express microglial markers and induce transcription of inflammatory cytokines and NFB pathway activation with poly(I:C) treatment which is modulated by *SH3RF3* knockdown. (A,B) Comparison of human and mouse brain scRNA‐seq data from a published database (brain RNAseq.org) shows species‐specific differential of *SH3RF3* expression across various brain cell types. Human fetal astrocytes and adult microglia express the highest levels of *SH3RF3* transcripts (A), whereas in the mouse brain, neurons express the highest level of *SH3RF3* transcripts (B). (C) Characterization of iMGLs with flow cytometry showing cell stained for microglial markers CD45 and CD11b, CD45^+^/CD11b^+^ double positive cells are compared with unstained control. (D) Immunostaining for microglial markers IBA1 (green) and P2RY12 (red) for iMGLs, DAPI (blue) showed in merge is used as a nuclear stain. (E) Western blot analysis of phosphorylated p105 and total p105 with 6 h poly(I:C) treatment with non‐targeting siRNA and *SH3RF3* siRNA in iMGLs. Representative blots and quantification is presented. Data are presented as mean ± SEM with *N* = 3 from two independent experiments. Comparisons are made using a mixed‐effects model with treatment group and differentiation as fixed factors, followed by Tukey's multiple comparisons test to determine *p*‐values for the main effect of treatment group (Control si ± poly(I:C) vs. SH3RF3 si ± poly(I:C)), **< 0.01.

**Figure S3:** Time course of cytokine transcription and NFκB pathway activation. (A) Recombinant monomeric Aβ42 was oligomerized overnight and used for experiments the following day. 200 ng of oligomers were run on a 4%–20% bis‐tris gel and transferred onto nitrocellulose membrane to probe against amyloid‐β (6E10 antibody). Oligomers are produced with a size consistent with trimers although higher order species are also present. (B) qPCR analysis of *IL1β*, *IL6*, and *TNFα* with 5 μM oligomeric amyloid‐β42 (oAβ) treatment in iMGLs at 6, 12, 24, and 48 h compared with vehicle control (F12). Data are normalized to *GAPDH* expression and presented as mean ± SEM with *N* = 3. (C) Western blot of phosphorylated and total p65 in iMGLs treated with vehicle (F12) 1 μM oAβ, 5 μM oAβ, or 25 μg/mL poly(I:C) for 3 h. Data are presented as mean ± SEM with *N* = 3. Comparisons are made by ordinary one‐way ANOVA followed by Tukey's multiple comparison test, **< 0.01, NS. (D) Western blot of phosphorylated and total p105 treated with vehicle or oAβ42 for 3 h treatment in presence of non‐targeting siRNA or *SH3RF3* siRNA in iMGLs. A representative blot and quantification for visible bands on oAβ42 conditions are shown. Data are presented as mean ± SEM with *N* = 3 from two independent experiments. Comparison of control Si with oAβ42 and *SH3RF3* Si with oAβ42 was analyzed via an unpaired *t*‐test, **< 0.01.

**Figure S4:** *PSEN1* ^G206A^ microglia exhibit a diminished inflammatory transcriptional response when stimulated with oAβ42. qPCR analysis of inflammatory cytokines IL1β, IL6, and TNFα upon vehicle or 5 μM oAβ42 treatment for 6 h in isogenic control and *PSEN1* ^G206A^ iMGLs. Data are presented as mean ± SEM by normalizing gene of interest with respective loading control *GAPDH* for *N* = 3 biological replicates per experiment for three independent experiments (*N* = 9). Statistical comparison between each group was examined using an unpaired *t*‐test. **< 0.01, ****< 0.0001, ns = not significant.

**Table S1:** Replications: Demographic and clinical characteristics of LOAD data, 4060 EFIGAs and 2058 WHICAPs.

**Table S2:** Differential gene expression of AD cases versus controls in Caribbean Hispanic, stratified by their *PSEN1* status.

## Data Availability

The data that support the findings of this study are available from the corresponding author upon reasonable request.
