# Supplementary material for: Reduced SH3RF3 May Protect Against Alzheimer's Disease by Lowering Microglial Pro‐Inflammatory Responses via Modulation of JNK and NFkB Signaling
Source: Glia. 2026 May 27;74(7):e70165. doi: 10.1002/glia.70165 (PMC13557802; doi:10.1002/glia.70165)
Supplement: Supplementary file 2 [file GLIA-74-e70165-s003.docx]

**Table S2. Differential gene expression of AD cases vs. controls in Caribbean Hispanic, stratified by their PSEN1 status.**
